# Metformin activates chaperone-mediated autophagy and improves disease pathologies in an Alzheimer disease mouse model

**DOI:** 10.1007/s13238-021-00858-3

**Published:** 2021-07-21

**Authors:** Xiaoyan Xu, Yaqin Sun, Xufeng Cen, Bing Shan, Qingwei Zhao, Tingxue Xie, Zhe Wang, Tingjun Hou, Yu Xue, Mengmeng Zhang, Di Peng, Qiming Sun, Cong Yi, Ayaz Najafov, Hongguang Xia

**Affiliations:** 1 Department of Biochemistry & Research Center of Clinical Pharmacy of The First Affiliated Hospital, Zhejiang University School of Medicine, Hangzhou, 310058 China; 2 Liangzhu Laboratory, Zhejiang University Medical Center, Hangzhou, 311121 China; 3 Department of Cell Biology, Harvard Medical School, Boston, MA 02115 USA; 4 Interdisciplinary Research Center on Biology and Chemistry, Shanghai Institute of Organic Chemistry, Chinese Academy of Sciences, Shanghai, 201203 China; 5 College of Pharmaceutical Sciences, Hangzhou Institute of innovative Medicine, Zhejiang University, Hangzhou, 310058 China; 6 Key Laboratory of Molecular Biophysics of Ministry of Education, Hubei Bioinformatics and Molecular Imaging Key Laboratory, College of Life Science and Technology, Huazhong University of Science and Technology, Wuhan, 430074 China

**Keywords:** chaperone-mediated autophagy, Metformin, TAK1, IKKα/β, Hsc70, APP, Alzheimer’s disease

## Abstract

Chaperone-mediated autophagy (CMA) is a lysosome-dependent selective degradation pathway implicated in the pathogenesis of cancer and neurodegenerative diseases. However, the mechanisms that regulate CMA are not fully understood. Here, using unbiased drug screening approaches, we discover Metformin, a drug that is commonly the first medication prescribed for type 2 diabetes, can induce CMA. We delineate the mechanism of CMA induction by Metformin to be via activation of TAK1-IKKα/β signaling that leads to phosphorylation of Ser85 of the key mediator of CMA, Hsc70, and its activation. Notably, we find that amyloid-beta precursor protein (APP) is a CMA substrate and that it binds to Hsc70 in an IKKα/β-dependent manner. The inhibition of CMA-mediated degradation of APP enhances its cytotoxicity. Importantly, we find that in the APP/PS1 mouse model of Alzheimer’s disease (AD), activation of CMA by Hsc70 overexpression or Metformin potently reduces the accumulated brain Aβ plaque levels and reverses the molecular and behavioral AD phenotypes. Our study elucidates a novel mechanism of CMA regulation via Metformin-TAK1-IKKα/β-Hsc70 signaling and suggests Metformin as a new activator of CMA for diseases, such as AD, where such therapeutic intervention could be beneficial.

**Supplementary Information:**

The online version contains supplementary material available at 10.1007/s13238-021-00858-3.

## Introduction

Chaperone-mediated autophagy (CMA) is one of three types of autophagy and has been identified only in birds and mammals (Gough and Fambrough 1997). CMA has been linked to many cellular processes, including metabolism, DNA repair, and T cell activation (Schneider et al. 2014; Valdor 2014; Kaushik and Cuervo 2015; Park et al. 2015; Zhang 2021). The selectivity of CMA is due to the requirement for its substrates to contain the Lys-Phe-Glu-Arg-Gln (KFERQ) motif (Dice et al. 1986; Dice 1990; Wing et al. 1991). The CMA substrate proteins are recruited to the lysosomes by heat shock protein family A (Hsp70) member 8 (Hsc70), which recognizes the KFERQ motifs and delivers the substrates to the lysosomal membrane (Chiang et al. 1989; Agarraberes and Dice 2001). Lysosomal associated membrane protein 2A (Lamp2a), a lysosomal receptor, mediates the translocation of CMA substrates into the lysosomal lumen, a process that is assisted by a luminal resident form of Hsc70 (Cuervo and Dice 1996; Agarraberes et al. 1997). The mechanistic regulation of CMA is not fully understood. No protein kinase has yet been reported to directly phosphorylate and regulate Hsc70 activity.

Dysregulation of CMA has been linked to neurodegeneration, cancer, aging, metabolic regulation, and T cell response (Kon 2011; Cuervo and Wong 2014; Schneider et al. 2014; Valdor 2014; Schneider 2015; Xilouri 2016). Therefore, a better understanding of the mechanistic regulation of CMA may pave the way for future therapeutic approaches for treating human diseases where CMA is dysregulated. However, the lack of a safe and effective drug that can selectively activate CMA has hampered research into the therapeutic feasibility of CMA-activating strategies.

Alzheimer’s disease (AD), the most common neurodegenerative disorder, is the leading cause of dementia and is characterized by a progressive neuronal loss with disease-defining Aβ peptide oligomerization and hyperphosphorylated Tau pathologies (Guo et al. 2012; Lane et al. 2018; Tiwari et al. 2019). CMA activation is suggested as a potential therapeutic strategy for AD due to its ability to decrease Tau protein levels (Scrivo et al. 2018). However, AD-associated forms of Tau aggregate and suppress CMA by binding to Lamp2a and disrupting its lysosomal translocation (Wang 2009). The role of CMA in regulating Aβ levels has not been described.

Aβ peptides are derived from amyloid precursor protein (APP) through sequential cleavages by β- and γ-secretases (Wang 2017). Inhibiting APP degradation is known to promote AD pathogenesis by facilitating Aβ production (Yang et al. 2013). Growing evidence indicates that aberrant modifications and trafficking of APP play crucial roles in AD pathogenesis by dysregulating APP processing and Aβ generation (Wang 2017; Lane et al. 2018). Therefore, pharmacologic facilitation of APP clearance could be a potential therapeutic strategy to treat AD.

Metformin is an oral drug widely used in the treatment of type 2 diabetes. It has been used with an excellent safety record for over 60 years (Diabetes Prevention Program Research, G. Long-term safety, tolerability, and weight loss associated with metformin in the Diabetes Prevention Program Outcomes Study 2012). Metformin has been shown to play a beneficial role in many diseases such as cancers (Gandini 2014), cardiovascular diseases (Lamanna et al. 2011), liver diseases (Bhat et al. 2015), obesity (Breining 2018) and renal diseases (Neven 2018). In addition, some studies have shown that Metformin can inhibit chronic inflammation (Tizazu 2019), anti-aging (Barzilai et al. 2016) and may preserve cognitive function (Ng 2014). Metformin induced activation of AMP activated protein kinase (AMPK), an energy sensor, contributed to part of the mechanism, but could not explain all the effects. The idea that Metformin exerts its pleiotropy through novel mechanisms is now increasingly supported.

In this study, we have screened 2,197 FDA-approved drugs or drug candidates and identified Metformin as a novel activator of CMA. We demonstrate that Metformin activates IKKα/β kinases, which in turn phosphorylate Hsc70 at Ser85 and activate it. We also find that APP is a CMA substrate and show that Metformin reduces Aβ levels and improves cognitive impairment in the APP/PS1 mouse model of AD. Finally, we find that AAV-mediated overexpression of Hsc70 in the hippocampus of APP/PS1 mice also significantly alleviates AD pathologies. Our findings identify Metformin as a novel inducer of CMA, discover the first reported mechanism of CMA activation downstream of TAK1-IKKα/β signaling, and suggest that CMA activation to degrade Aβ could be a putative therapeutic strategy for AD.

## Results

### Identification of CMA activators by high-throughput screening

**Figure 1:**
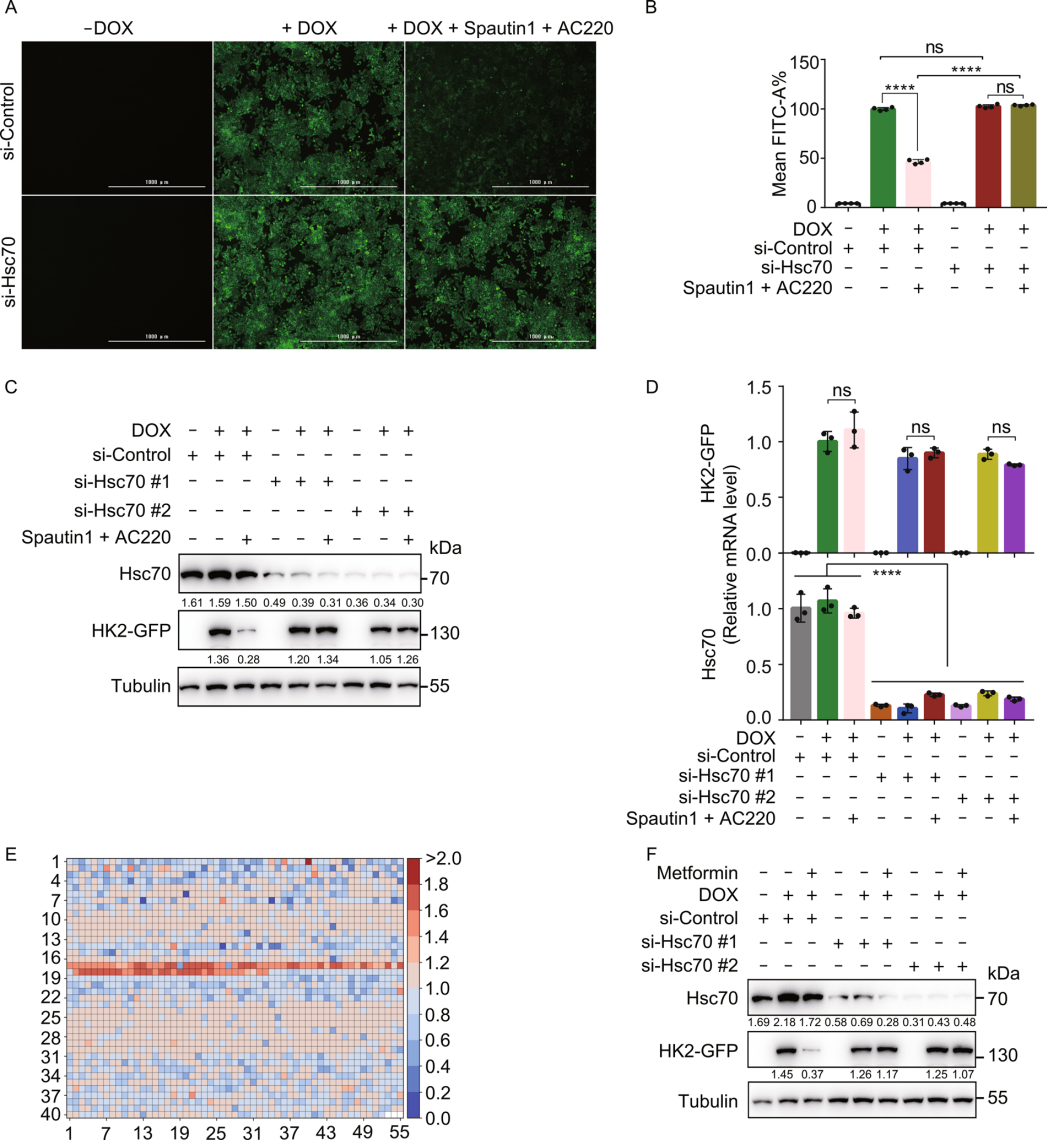
Identification of CMA-inducing drugs by high-throughput screening. (A) 293THK cells were pretreated with or without 1 μg/mL DOX and transfected with siRNA targeting Hsc70 for 48 h, treated with Spautin-1 (10 μmol/L) and AC220 (2 μmol/L) for another 12 h, fluorescence of HK2-GFP was imaged by fluorescence microscopy. Scale bar, 1000 μm. (B) The fluorescence intensity of (A) were quantified from four independent experiments, Mean FITC-A% represents the average fluorescence intensity of cells (data represents mean ± SD; *n* = 4, ****P < 0.0001, *t*-test). (C) 293THK cells were treated as in **a**, the substrate protein (HK2-GFP) was detected by Western blot. (D) 293THK cells were treated as in (A). Total RNA was extracted by FastPure® Cell/Tissue Total RNA Isolation Kit, mRNA levels of GFP and Hsc70 were analyzed by qPCR (data represents mean ± SD; *n* = 3, ****P < 0.0001, *t*-test). (E) 293THK cells were pretreated with or without 1 μg/mL DOX for 24 h, incubated with 2,197 FDA-approved drugs or drug candidates for 24 h, and the HK2-GFP fluorescence was analyzed by flow cytometry and compared with the fluorescence of cells treated with DMSO. The results were presented in the form of a heatmap. Red and blue colors represent the degree of increase and decrease of HK2-GFP levels, respectively, following drug treatments. (F) 293THK cells were pretreated with or without 1 μg/mL DOX, transfected with siRNA (two different sequences #1 and #2) of Hsc70 for 48 h, treated with or without Metformin for another 12 h, cell lysates were analyzed by Western blot using anti-Hsc70, anti-GFP, and anti-Tubulin antibodies

Hexokinase 2 (HK2), a key enzyme involved in glucose metabolism, has been shown to be a CMA substrate (Xia 2015). In order to establish a CMA reporter system suitable for high-throughput screening, we generated a HEK293T cell line stably expressing HK2-GFP in a doxycycline (DOX)-inducible manner (henceforth, 293THK). Treating cells with a combination of autophagy inhibitor Spautin-1 and Fms-like tyrosine kinase-3 (Flt3) inhibitor AC220 has been previously shown to induce HK2 degradation via CMA (Vakifahmetoglu-Norberg 2013; Xia 2015). Consistently, a major decrease of GFP fluorescence and HK2-GFP levels were induced by this treatment, and this decrease was rescued by knockdown of the CMA mediator Hsc70 (Figs. 1A–C and S1A). The mRNA levels of HK2-GFP were not affected by these treatments, confirming that the observed decrease was due to protein degradation (Fig. 1D). These findings established that the 293THK cell line is a functional reporter system for monitoring CMA activity.

To identify CMA activators, we screened 2,197 FDA-approved drugs or drug candidates using 293THK cells and high-throughput flow cytometry and identified 195 compounds that induced a significant decrease in HK2-GFP fluorescence (Fig. 1E). Using these hits, we performed a second round of screening, but in the presence or absence of Lamp2a knockdown (to block CMA) and identified 38 compounds that induce a decrease in HK2-GFP levels only in the absence of Lamp2a knockdown (Fig. S1B). Notably, Metformin, a drug frequently prescribed for type 2 diabetes, was among these 38 compounds.

### Metformin activates chaperone-mediated autophagy

**Figure 2:**
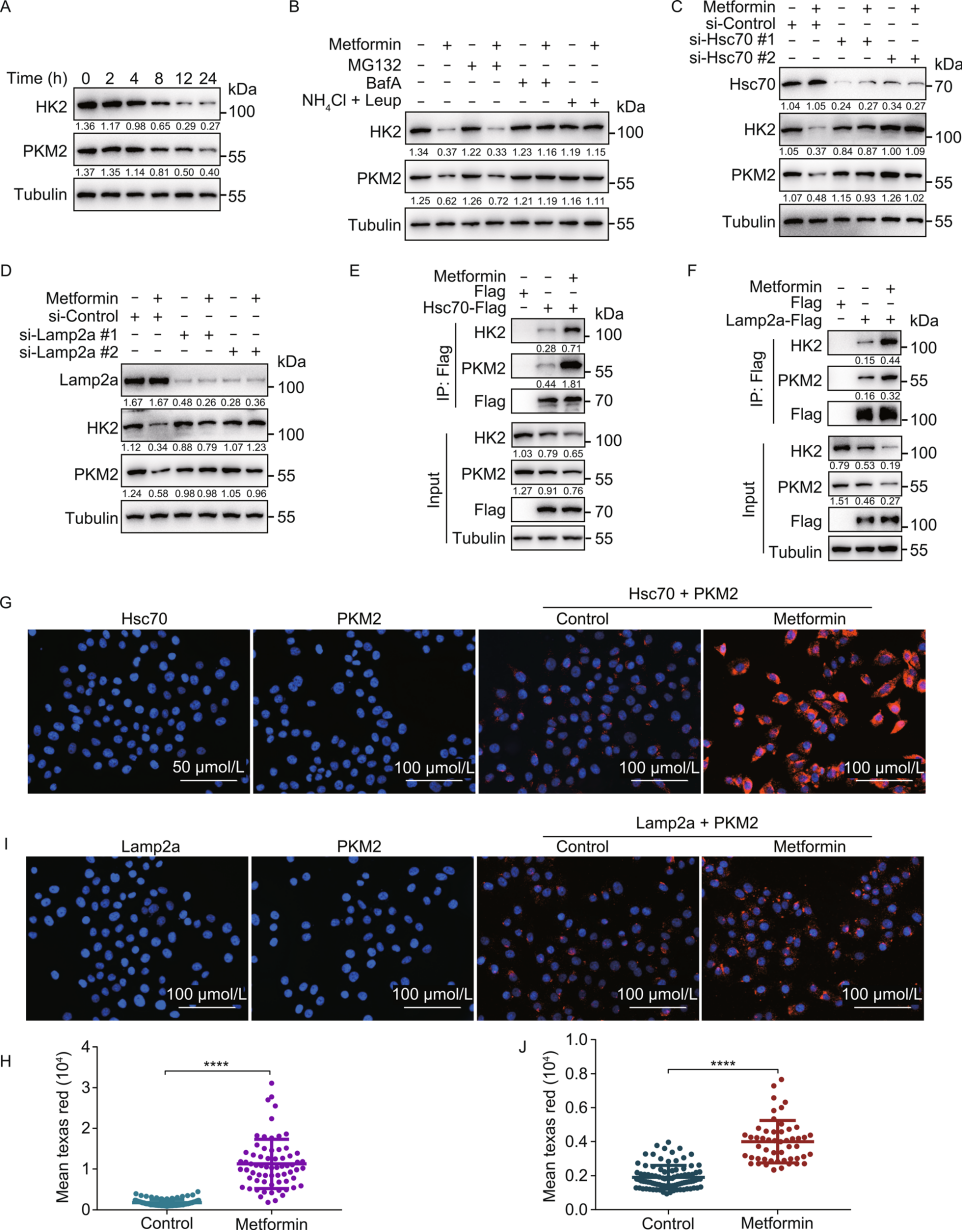
Metformin activates chaperone-mediated autophagy. (A) H4 cells were treated with 20 mmol/L Metformin for 2, 4, 8, 12, and 24 h. Cell lysates were immunoblotted with indicated antibodies. (B) H4 cells were treated with 20 mmol/L Metformin with or without MG132 (10 μmol/L), Bafilomycin A1 (100 nmol/L), NH_4_Cl (20 mmol/L), Leupeptin (100 nmol/L), E-64D (10 μmol/L) for 12 h. Cell lysates were immunoblotted with indicated antibodies. (C and D) H4 cells were transfected with indicated siRNAs (#1 and #2 represent two different sequences) for 48 h, treated with or without 20 mmol/L Metformin for another 12 h. Cell lysates were immunoblotted with indicated antibodies. (E) HEK293T cells were transfected with Hsc70-Flag for 24 h, treated with or without 20 mmol/L Metformin for another 6 h, the interaction between HK2, PKM2, and Hsc70 were analyzed by immunoprecipitation. (F) HEK293T cells were transfected with Lamp2a-Flag for 24 h, treated with or without 20 mmol/L Metformin for another 6 h, the interaction between HK2, PKM2, and Lamp2a were analyzed by immunoprecipitation. (G) H4 cells were treated with or without 20 mmol/L Metformin for 6 h, PLA assay for endogenous Hsc70 and PKM2 was analyzed by fluorescence microscopy. Scale bar, 100 μm. (H) Quantification of the fluorescence intensity of Texas Red from (G) (data represents mean ± SD; ****P < 0.0001, one-way ANOVA). (I) H4 cells were treated with or without 20 mmol/L Metformin for 6 h, PLA assay for endogenous Lamp2a and PKM2 was analyzed by fluorescence microscopy. Scale bar, 100 μm. (J) Quantification of the fluorescence intensity of Texas Red from (I) (data represents mean ± SD; ****P < 0.0001, one-way ANOVA)

As shown in Fig. 1F, Metformin treatment induced a decrease in HK2-GFP protein levels, and knockdown of Hsc70 rescued this effect, indicating a CMA-dependent decrease in the protein levels. We confirmed that Metformin did not affect the mRNA levels of HK2-GFP (Fig. S1C). We also detected the dose course curve of Metformin on degradation of HK2-GFP, the degradation of HK2-GFP was accelerated following the increase dose of Metformin (Fig. S1D). We also measured the half-life of HK2-GFP, as shown in Fig. S1E, the degradation of HK2-GFP which treated with Metformin was accelerated compared with control. These results indicated that the effects of Metformin on HK2-GFP was through degradation. Metformin also induced degradation of two endogenous CMA substrates—HK2 and PKM2 (pyruvate kinase isozyme type M2), at both 20 mmol/L and 20 µmol/L doses of the drug (Figs. 2A and S2A). We chose these two doses due to the robustness of the 20 mmol/L dose and due to the clinical relevance of the 20 µmol/L dose, as the Metformin serum concentrations in patients receiving this drug are ~20 µmol/L (Graham 2011).

Metformin-induced degradation of HK2 and PKM2 was blocked by lysosomal inhibitors (E-64D, Bafilomycin A1 and Leupeptin + NH_4_Cl) but not by the proteasome inhibitor MG132 which confirming that the degradation is lysosome-dependent (Figs. 2B and S2B). Knockdown of Hsc70 and Lamp2a blocked the degradation of endogenous HK2 and PKM2, following both 20 mmol/L (Fig. 2C and 2D) and 20 μmol/L (Fig. S2C and S2D) doses of Metformin treatment, indicating a CMA-dependent degradation of these proteins. Additionally, knockdown of Hsc70 also rescued Metformin-induced decrease of HK2-GFP fluorescence and HK2-GFP protein levels (Fig. S2E and S2F). Moreover, Metformin induced interaction of endogenous HK2 and PKM2 with Hsc70-Flag (Figs. 2E and S2G) and Lamp2a-Flag (Figs. 2F and S2H), as judged by pull-down experiments. These findings were also confirmed by a proximity ligation assay (PLA) performed for endogenous Hsc70 (Fig. 2G and 2H) and endogenous Lamp2a (Fig. 2I and 2J). We also detected whether macroautophagy were involved in the degradation of HK2 and PKM2. Using ATG5 knockout HEK293 cells and ATG8 knockout Hela cells, we found that knockout of ATG5 and ATG8 did not block the degradation of HK2 and PKM2 under Metformin treatment (Fig. S2I–L).

Overall, these experiments confirm that Metformin activates CMA, as it triggers degradation of endogenous CMA substrates in a lysosome-, Hsc70-, and Lamp2a-dependent manner and concurrently increases their interactions with Hsc70 and Lamp2a.

### Metformin activates CMA via Ser85 phosphorylation of Hsc70

**Figure 3:**
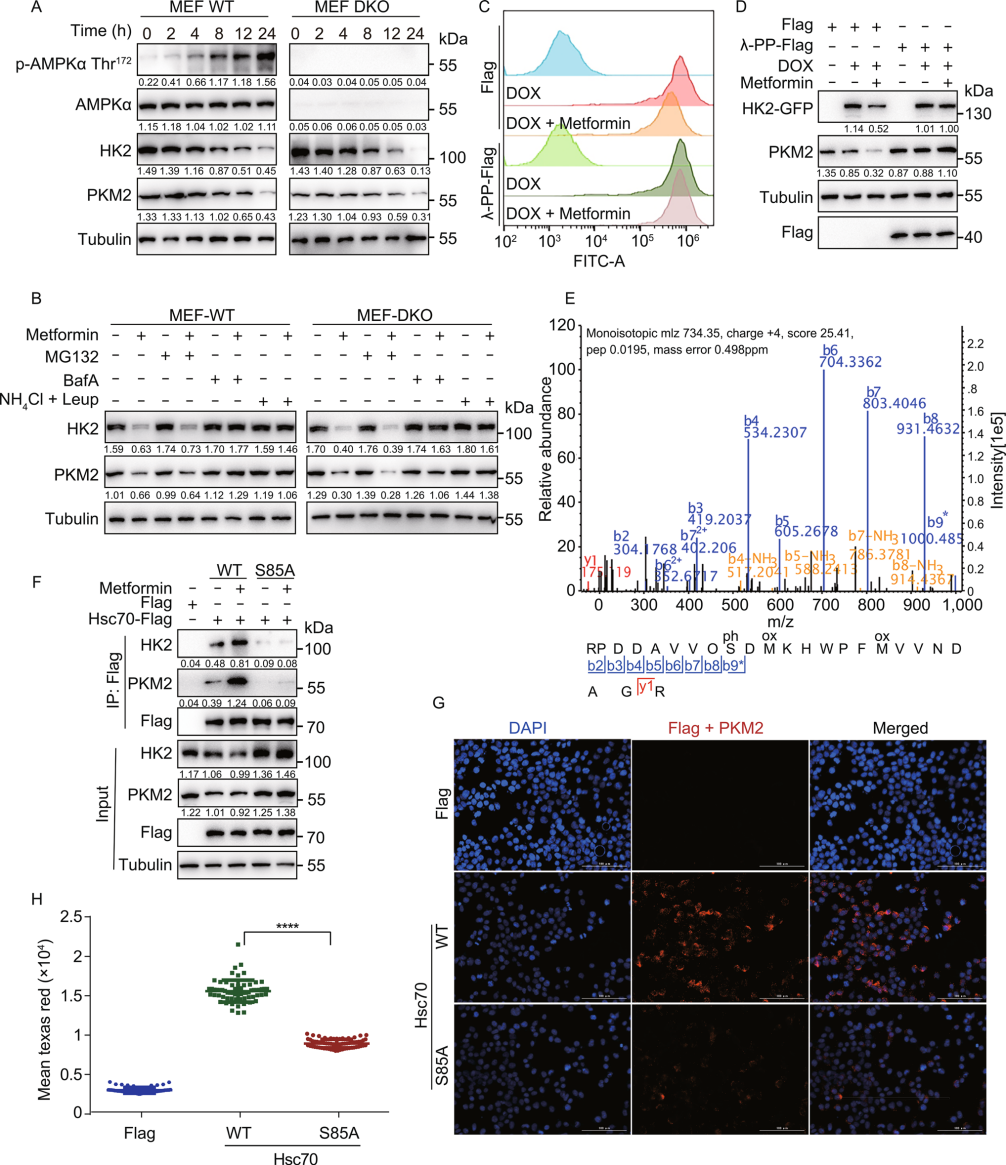
Metformin activates chaperone-mediated autophagy by inducing Hsc70 phosphorylation at Ser85. (A) AMPK wild-type (WT) and α1/α2 double knockout (DKO) MEF cells were treated with 20 mmol/L Metformin for 2, 4, 8, 12, and 24 h. Cell lysates were immunoblotted with indicated antibodies. (B) AMPK WT and DKO MEF cells were treated with 20 mmol/L Metformin with or without MG132 (10 μmol/L), Bafilomycin A1 (100 nmol/L), NH_4_Cl (20 mmol/L), Leupeptin (100 nmol/L) for 12 h. Cell lysates were immunoblotted with indicated antibodies. (C) 293THK cells were pretreated with or without 1 μg/mL DOX, transfected with Flag or PP-Flag (λ-PPase) plasmids for 24 h, treated with or without 20 mmol/L Metformin for another 12 h. The fluorescence of HK2-GFP was analyzed by flow cytometry. (D) 293THK cells were treated as shown in (C). Cell lysates were immunoblotted with indicated antibodies. (E) HEK293T cells were transfected with Hsc70-Flag for 24 h, treated with or without Metformin (20 mmol/L) for another 8 h, Hsc70 was immunoprecipitated using Flag-agarose beads and analyzed by MS-MS. (F) HEK293T cells were transfected with indicated Hsc70 plasmids for 24 h, treated with or without 20 mmol/L Metformin for another 6 h, the interaction between Hsc70 and HK2 or PKM2 was analyzed by co-immunoprecipitation. (G) HEK293T cells were transfected with Hsc70 WT or Hsc70 S85A plasmids for 24 h, PLA assay for Hsc70 and PKM2 was performed using fluorescence microscopy. Scale bar, 100 μm. (H) Quantification of the mean fluorescence intensity of Texas Red from (G) (data represents mean ± SD; ****P < 0.0001, one-way ANOVA).

Metformin is a known inducer of AMPK activation (Hardie et al. 2012; Zhang 2016). Using AMPKα1/α2 double knockout (DKO) mouse embryonic fibroblast (MEF) cells, we found that the degradation of endogenous HK2 and PKM2 following Metformin treatment was independent of AMPK (Figs. 3A and S3A). However, Metformin-induced degradation of the CMA substrates HK2 and PKM2 was lysosome-dependent and proteasome-independent in these cells (Fig. 3B), as in H4 cells (Fig. 2B).

We found that Metformin-induced decrease of HK2-GFP fluorescence and protein levels were rescued by overexpression of λ-phosphatase, suggesting that a phosphorylation event is responsible for the degradation of HK2-GFP (Figs. 3C, 3D, S3B, and S3C). We subjected Hsc70-Flag immunoprecipitated from control versus Metformin-treated cells to phosphorylation analysis by mass spectrometry and found that Ser85 phosphorylation was induced by the treatment (Fig. 3E). Interestingly, the Metformin-induced interaction between Hsc70 and the CMA substrate proteins was significantly decreased by S85A mutation of Hsc70 (Figs. 3F and S3D), the Hsc70-PKM2 interaction detected by PLA was also blocked by the S85A mutation of Hsc70 (Fig. 3G and 3H). We also tested whether S85A mutation of Hsc70 influence CMA with or without Lamp2a knockdown in H4 and HKE293T cells. Overexpression of Hsc70-WT but not Hsc70-S85A decreased protein levels of HK2 and PKM2 and knockdown of Lamp2a blocked these degradation (Fig. S3E and S3F). These results suggest that the Metformin-induced activation of CMA may involve phosphorylation of Hsc70 at Ser85, promotes interaction of Hsc70 with the CMA substrate proteins. These findings reveal not only an unexpected effect downstream of Metformin treatment but also a previously unknown regulatory input into Hsc70 and CMA, as well as the importance of Hsc70 phosphorylation at Ser85 to promote Hsc70 interaction with CMA substrates.

### Metformin activates TAK1-IKKα/β signaling

We generated a phospho-specific antibody that recognizes Hsc70 phosphorylated at Ser85 (p-Hsc70 Ser^85^). A strong signal was detected in HEK293T cells transfected with WT, but not S85A Hsc70, indicating that the antibody specifically recognizes Hsc70 phosphorylated at Ser85 (Fig. S4A). We also found that Metformin treatment induced the phosphorylation of Hsc70 in a time-dependent manner (Fig. S4B–E). These results indicated that the phosphorylation of Hsc70 at Ser85 is important for the activation of CMA.

**Figure 4:**
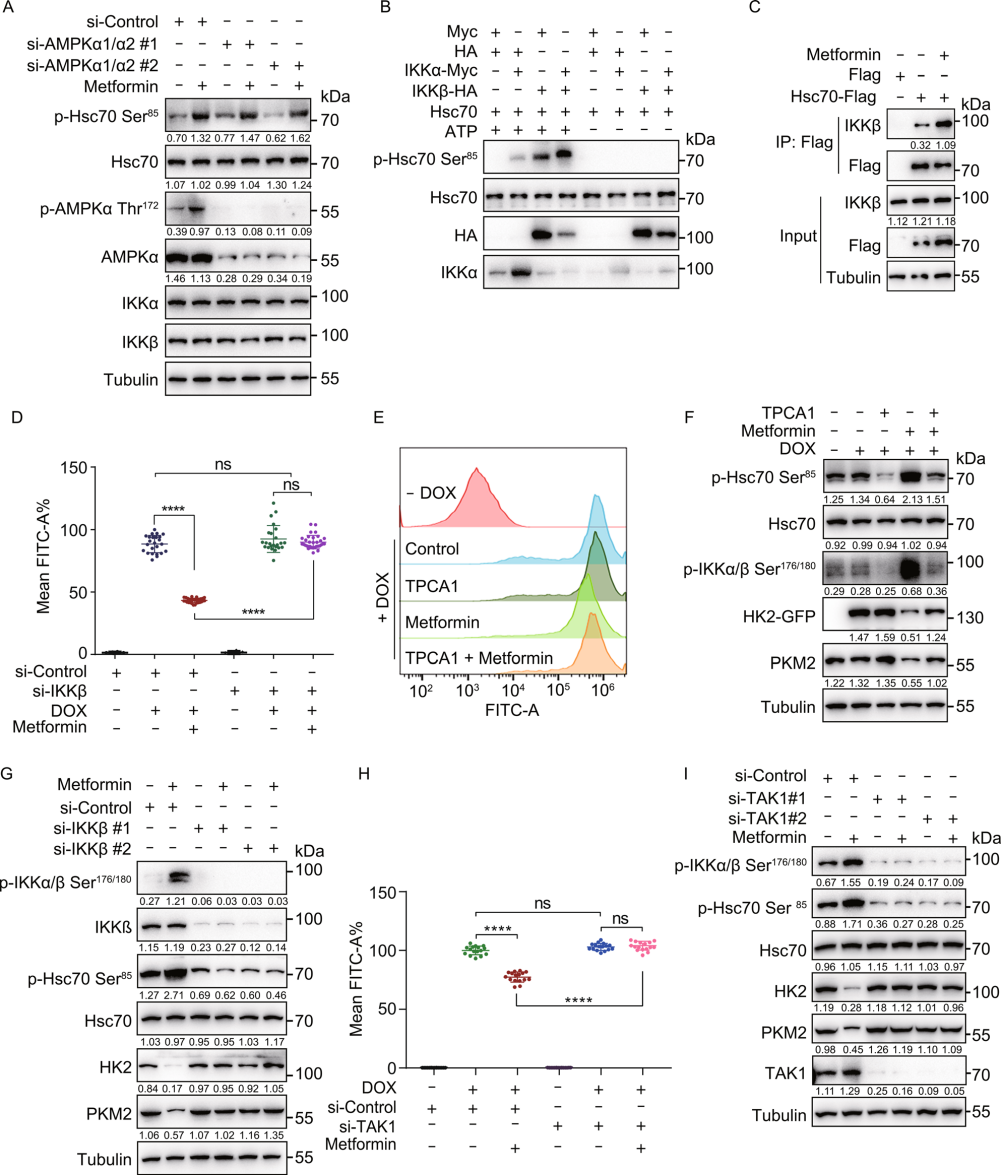
Metformin activates IKKα/β kinases to promote Hsc70 phosphorylation at Ser85 in a TAK1-dependent and AMPK-independent manner. (A) HEK293T cells were transfected with indicated siRNA for 48 h, treated with or without 20 mmol/L Metformin for another 12 h. Cell lysates were immunoblotted with indicated antibodies. (B) *In vitro* kinase reactions using purified IKKα, IKKβ, and Hsc70. The reactions were immunoblotted with indicated antibodies. (C) HEK293T cells were transfected with Hsc70-Flag for 24 h, treated with or without Metformin (20 mmol/L) for another 6 h, and the interaction between IKKβ and Hsc70 was detected by co-immunoprecipitation. (D) 293THK cells were pretreated with or without 1 μg/mL DOX, transfected with siRNA (#1 and #2 represent two different sequences) of IKKβ for 48 h, treated with or without 20 mmol/L Metformin for another 12 h, fluorescence of HK2-GFP was analyzed by flow cytometry (data represents mean ± SD; ****P < 0.0001, one-way ANOVA). (E) 293THK cells were pretreated with or without 1 μg/mL DOX, treated with 20 mmol/L Metformin with or without 5 μmol/L TPCA1 for 12 h, fluorescence of HK2-GFP was analyzed by flow cytometry. (F) 293THK cells were treated as shown in (E). Cell lysates were immunoblotted with indicated antibodies. (G) H4 cells were transfected with siRNA (#1 and #2 represent two different sequences) of IKKβ for 48 h, treated with or without 20 mmol/L Metformin for another 12 h. Cell lysates were immunoblotted with indicated antibodies. (H) 293THK cells were pretreated with or without 1 μg/mL DOX, transfected with siRNA (#1 and #2 represent two different sequences) of TAK1 for 12 h, treated with or without 20 μmol/L Metformin for another 48 h, the fluorescence of HK2-GFP was analyzed by flow cytometry (data represents mean ± SD; ****P < 0.0001, one-way ANOVA). (I) H4 cells were transfected with siRNA (#1 and #2 represent two different sequences) of TAK1 for 12 h, treated with or without 20 μmol/L Metformin for another 48 h. Cell lysates were immunoblotted with indicated antibodies.

Consistent with the AMPK-independent nature of the Metformin-induced CMA activation (Fig. 3A), the phosphorylation of Hsc70 at Ser85 was also independent of AMPK activation, as AMPKα1/α2 knockdown in HEK293T cells (Fig. 4A) and AMPKα1/α2 knockout in MEF cells (Fig. S3A) did not block it. We found that the Ser85 of Hsc70 is predicted to be a potential IKKα/β target (see Methods section). *In vitro* kinase assays using purified Hsc70 and IKKα/β revealed that Hsc70 is directly phosphorylated at Ser85 by both IKKα and IKKβ, and combination of IKKα and IKKβ further increases the phosphorylation of Hsc70 at Ser85 (Fig. 4B). The interaction between IKKα or IKKβ and Hsc70 was increased following Metformin treatment (Figs. 4C and S4F). These results suggest that IKKα/β phosphorylate Hsc70 at Ser85.

To further verify the upstream kinases of Hsc70 at Ser85 is IKKα/β, we knocked down IKKα and IKKβ in 293THK cells and treated with Metformin. Knockdown of IKKβ rescued Metformin-induced reduction of HK2-GFP fluorescence and protein levels (Figs. 4D and S5A–C). Knockdown of IKKα also showed the same results (Fig. S4G and S4H). Inhibition of IKKβ by TPCA1 also rescued the Metformin-induced decrease of HK2-GFP levels (Figs. 4E, 4F, S5D, and S5E). Furthermore, TPCA1 inhibited the Metformin-induced phosphorylation of Hsc70 at Ser85 and inhibited Metformin-induced degradation of PKM2 (Figs. 4F and S5E). Obviously, knockdown of IKKα or IKKβ reduced the p-Hsc70 Ser^85^ levels and prevented the degradation of endogenous HK2 and PKM2 (Figs. 4G, S4I, and S5F). Furthermore, overexpression of IKKα or IKKβ increased the levels of p-Hsc70 Ser^85^ (Fig. S4J and S5G). Overall, these experiments suggest that Metformin-induced phosphorylation of Hsc70 at Ser85 is mediated by IKKα and IKKβ.

TAK1 (transforming growth factor beta-activated kinase 1) is known to activate IKKα/β kinases via phosphorylation at their Ser176/180 residues (Wang 2001). Therefore, we tested whether Metformin-induced IKK and CMA activation are dependent on TAK1. We found that knockdown of TAK1 rescued the Metformin-induced decrease of HK2-GFP levels (Figs. 4H and S5H), the degradation of endogenous HK2 and PKM2, as well as induction of p-IKKα/β Ser^176/180^ and p-Hsc70 Ser^85^ (Fig. 4I). These findings suggest that Metformin triggers TAK1 activation, which subsequently phosphorylates and activates IKKα/β that phosphorylate Hsc70 to induce CMA.

In order to verify whether TAK1 is a direct target of Metformin for CMA activation, we detected whether Metformin has direct interaction with TAK1 by thermal shift assay *in vitro*. The addition of TAK1 inhibitor 5a-7-oxozeaenol (5z7) to TAK1 decreased its melting temperature (Tm) by 8.28 °C , however, Metformin had no effect (Fig. S5I), suggesting that there was no direct interaction between Metformin and TAK1. These results indicated that TAK1 is not the direct target of Metformin, however it involved in the activation of CMA by Metformin.

### APP is a CMA substrate

**Figure 5:**
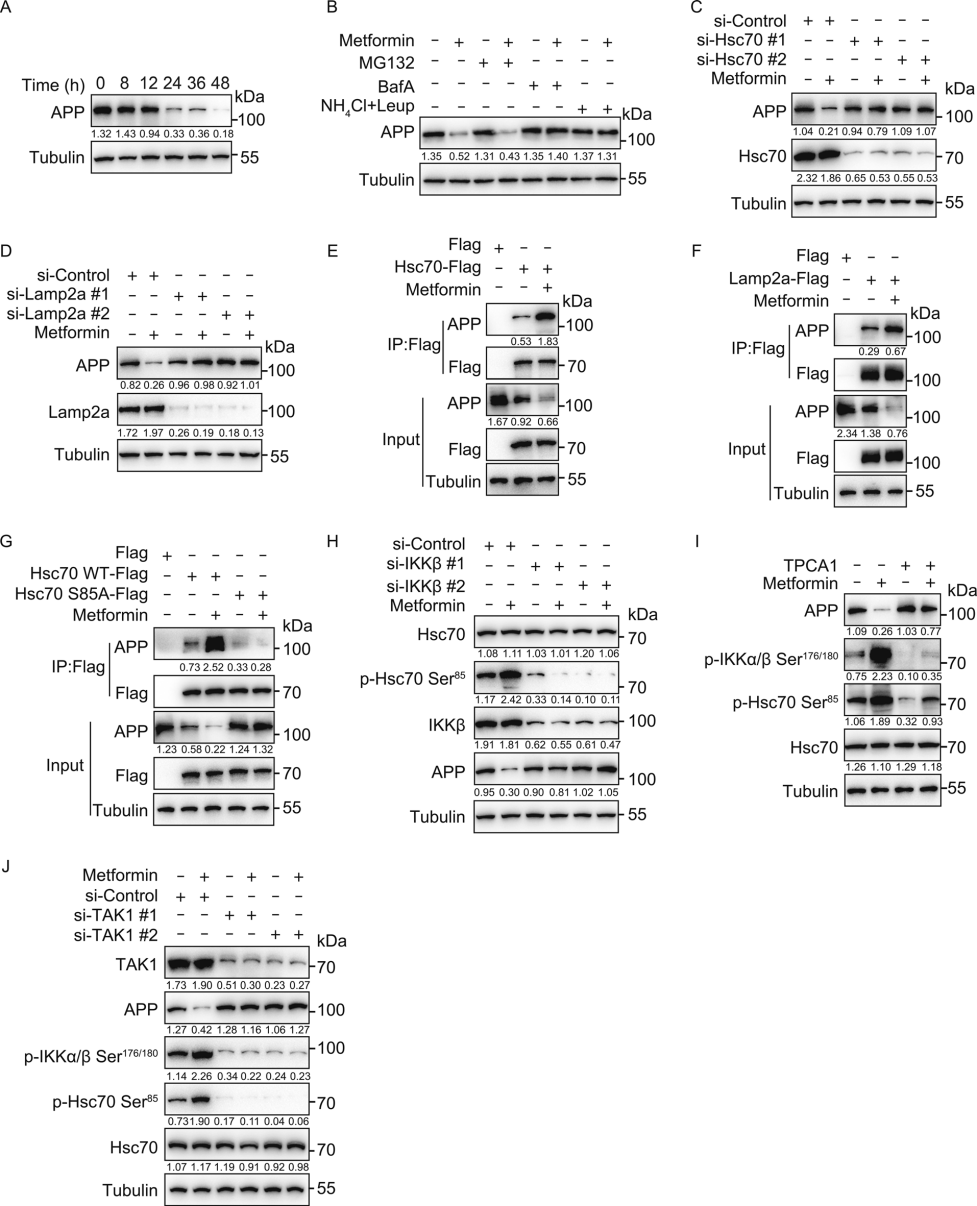
Metformin induces APP degradation through activation of chaperone-mediated autophagy. (A) SH-SY5Y cells were treated with Metformin (20 μmol/L) for 8, 12, 24, 36 and 48 h. Cell lysates were immunoblotted with indicated antibodies. (B) SH-SY5Y cells were treated with or without Metformin (20 μmol/L) for 36 h, with MG132 (10 μmol/L), Bafilomycin A1 (100 nmol/L), NH_4_Cl (20 mmol/L), Leupeptin (100 nmol/L) for another 12 h. Cell lysates were immunoblotted with indicated antibodies. (C and D) SH-SY5Y cells were transfected with siRNA (#1 and #2 represent two different sequences) of Hsc70 (C) or Lamp2a (D) for 12 h, treated with or without Metformin (20 μmol/L) for another 48 h, cell lysates were immunoblotted with indicated antibodies. si-Control: scrambled siRNA. (E and F) SH-SY5Y cells were transfected with Hsc70-Flag (E) or Lamp2a-Flag (F) for 24 h, treated with or without Metformin (20 μmol/L) for another 12 h, the interaction between APP and Hsc70 was analyzed by immunoprecipitation. (G) SH-SY5Y cells were transfected with Hsc70 WT-Flag or Hsc70 S85A-Flag for 24 h, treated with or without Metformin (20 μmol/L) for another 12 h, the interaction between APP and Hsc70 was analyzed by immunoprecipitation. (H) SH-SY5Y cells were transfected with siRNA (#1 and #2 represent two different sequences) of IKKβ for 12 h, treated with or without Metformin (20 μmol/L) for another 48 h. Cell lysates were immunoblotted with indicated antibodies. si-Control: scrambled siRNA. (I) SH-SY5Y cells were treated with Metformin (20 μmol/L) for 36 h, with or without TPCA1 (5 μmol/L) for another 12 h. Cell lysates were immunoblotted with indicated antibodies. (J) SH-SY5Y cells were transfected with siRNA (#1 and #2 represent two different sequences) of TAK1 for 12 h, treated with or without Metformin (20 μmol/L) for another 48 h. Cell lysates were immunoblotted with indicated antibodies. si-Control: scrambled siRNA.

CMA has been proposed to be a potential therapeutic avenue to treat Alzheimer’s disease (AD) because of the ability of CMA to degrade Tau. However, Tau aggregates are known to inhibit CMA. Whether APP can also be degraded by CMA is not known. Due to its importance in the pathogenesis of AD, we investigated whether APP can be targeted to degradation following CMA activation by Metformin. Notably, Metformin induced degradation of endogenous APP proteins in SH-SY5Y cells, in a lysosome-dependent (Fig. 5A and 5B), as well as Hsc70- and Lamp2a-dependent manner (Fig. 5C and 5D).

Metformin treatment also enhanced the interaction between endogenous APP and Hsc70-Flag, as well as Lamp2a-Flag (Fig. 5E and 5F). Consistent with the observation that Ser85 phosphorylation of Hsc70 is important for its interaction with CMA substrates, as shown in Fig. 5G, the interaction of Hsc70-Flag with APP was also blocked by the S85A mutation of Hsc70. Notably, inhibition and knockdown of IKKβ (Fig. 5H and 5I), as well as knockdown of TAK1 (Fig. 5J), blocked the Metformin-induced degradation of APP, concomitantly blocking Metformin-induced phosphorylation of IKKα/β at Ser176/180 and Hsc70 at Ser85. Collectively, these results indicate that APP is a CMA substrate, degradation of which can be induced by Metformin via activation of the TAK1-IKKα/β-Hsc70-CMA pathway.

We found that the amino acid sequence of APP contains three putative KFERQ-like CMA motifs that are strongly conserved across species (Fig. S6A and S6B). In order to verify that these motifs are involved in APP degradation through Metformin-activated CMA, we constructed APP mutants of the KFERQ-like motifs, as shown in Fig. S6A. The Metformin-induced degradation of APP was not blocked by mutation of any single KFERQ-like motif. However, mutation of two or especially three of the motifs prevented the degradation (Fig. S6C–E). Importantly, the interaction of APP with endogenous Hsc70 or Lamp2a was blocked when all three CMA motifs in APP (APP-M7) were mutated (Fig. S6F). These findings suggest that APP interacts with Hsc70 through the KFERQ-like motifs during activation of CMA.

### Metformin alleviates cytotoxicity of APP and Aβ via CMA-mediated degradation

APP upregulation can promote AD pathogenesis by facilitating Aβ production. SH-SY5Y cells can be used to detect the cell viability of overexpression of APP (Scuderi et al. 2014). We found that overexpression of APP-WT in SH-SY5Y cells reduced cell viability and that Metformin treatment rescued this cytotoxic effect. However, this Metformin-induced rescue was blocked by the mutations of the KFERQ-like motifs of APP (APP-M7), indicating that the effect of Metformin on cell viability depends on CMA-mediated degradation of APP (Fig. S6G). Similarly, transfection of Hsc70, but not Hsc70-S85A rescued the drop in cell viability following overexpression of APP, indicating that CMA-mediated degradation of APP can rescue the cytotoxicity effect due to its aberrantly high levels (Fig. S6H and S6I).

Notably, Metformin treatment also rescued cytotoxicity caused by overexpression of the disease-causing Swedish K595N/M596L APP mutant (Fig. S6J). Consistently, Metformin induced degradation of both wild-type and K595N/M596L APP (Fig. S6K). These results suggest that Metformin can prevent cytotoxicity induced by overexpression of APP and the disease-causing K595N/M596L APP mutant by targeting them to CMA-mediated degradation.

Aβ-induced neurotoxicity can be detected by the cell viability of PC12 cells incubated with Aβ (Fezoui 2000; Chromy 2003). Preventing Aβ-induced neurotoxicity has been proposed as a possible strategy for therapeutic intervention in AD. We found that Metformin blocked Aβ_25-35_-induced cytotoxicity in Hsc70- and Lamp2a-dependent manner in PC12 cells, suggesting the involvement of CMA in this effect (Fig. S7A and S7B). Notably, IKKβ inhibitor TPCA1 and knockdown of TAK1 also blocked this effect of Metformin, consistent with our findings that TAK1-IKKα/β signaling is important for Metformin-induced activation of CMA (Fig. S7C and S7D). These results suggest that the effect of Metformin on Aβ-induced toxicity is via the TAK1-IKKα/β-CMA signaling.

In order to differentiate at which extent Metformin promotes degradation of APP or alleviating of Aβ cytotoxicity via CMA or macroautophagy, we use siRNAs to target ATG5, and found that the alleviating of cytotoxicity which induced by overexpression of APP or Aβ by Metformin was not blocked by knockdown of ATG5 (Fig. S7E and S7G). Knockdown of ATG5 also did not block the degradation of APP under Metformin treatment (Fig. S7F).

Overall, our findings indicate that Metformin-induced CMA activation can induce degradation of APP, disease-causing Swedish K595N/M596L APP mutant and rescue the associated cytotoxicity in cell culture.

### Activation of CMA by Metformin or overexpression of Hsc70 ameliorates cognitive decline and amyloid pathologies in the APP/PS1 mouse model of AD

**Figure 6:**
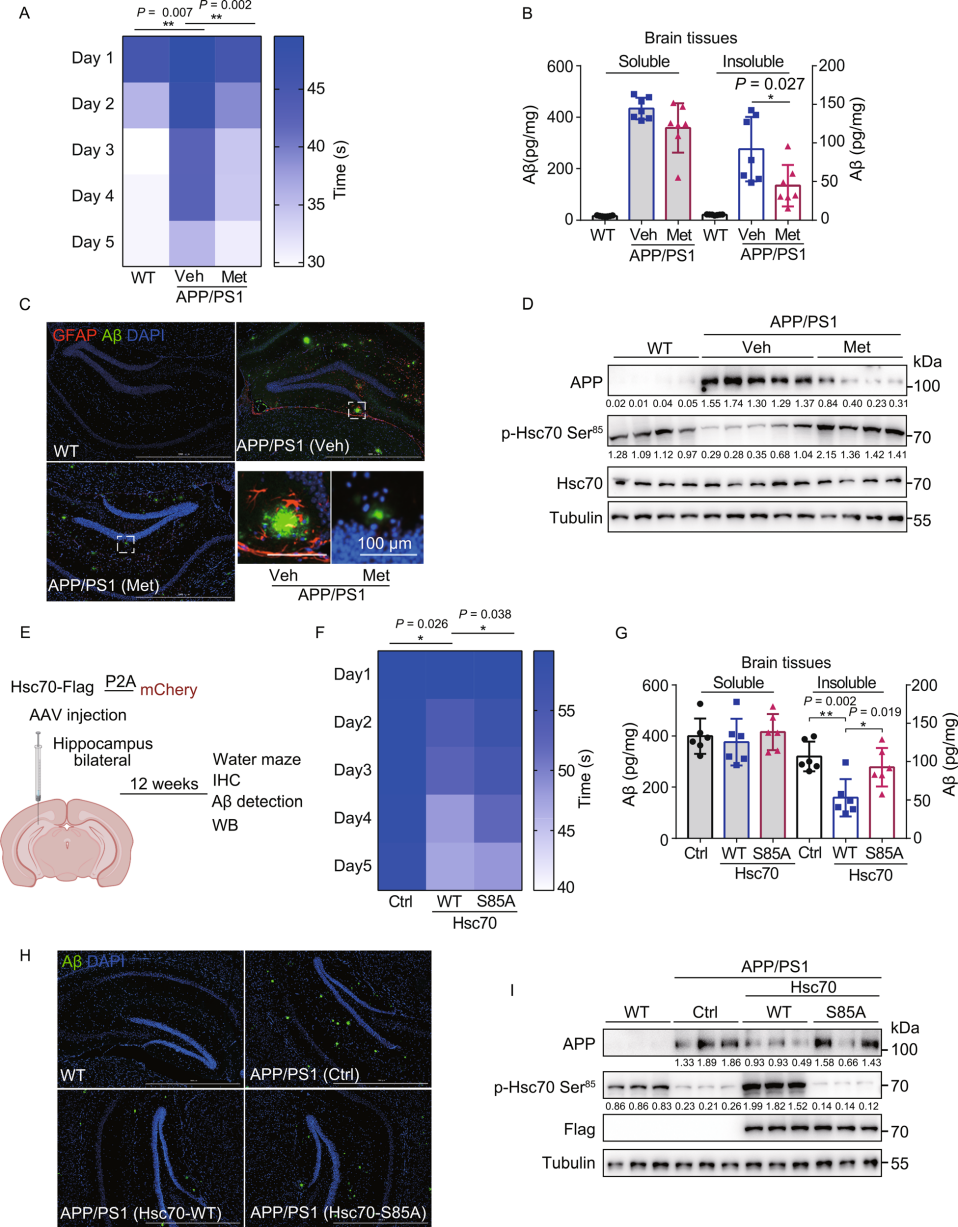
Chaperone-mediated autophagy activation by Metformin and overexpression of Hsc70 in the APP/PS1 mouse model of Alzheimer’s disease. (A) Wild-type or APP/PS1 mice were treated with either water (Veh) or Metformin (3 g/L) (Met) in drinking water for 12 weeks and were subjected to the Morris water maze (MWM) test. Latency to escape to a hidden platform (time to find the platform shown in the right panel) during a 5-day training period is shown (*n* = 7, mean ± SD; **P < 0.01, *t*-test). (B) Mice were treated as in (A), and brain tissues were analyzed for soluble and insoluble Aβ_1-42_ levels, using ELISA (mean ± SD; *P < 0.05, *t*-test). (C) Mice were treated as in (A) and immunohistochemistry of the hippocampus was performed to stain for amyloid plaques (6E10 antibody, green), astrocytes (GFAP antibody, red), and nuclei (DAPI, blue). Scale bar, 1000 μm; insets: scale bar, 100 μm. (D) Mice were treated as in (A) and mouse brain tissue lysates were immunoblotted with indicated antibodies (each band represents random independent mouse sample, *n* = 4 mice in WT, *n* = 5 mice in APP/PS1 (Veh) and *n* = 4 mice in APP/PS1 (Metformin)), the numbers under the blots represent the grayscale quantification normalized to tubulin. (E) Outline of the experimental design for the data shown in Fig. 6F–I. AAV-mCherry, AAV-Hsc70 WT-Flag-P2A-mCherry, or AAV-Hsc70 S85A-Flag-P2A-mCherry were injected into the hippocampus of 16-week-old APP/PS1 mice and behavioral tests and immunostaining were performed 12 weeks after the injections. (F) Mice were treated as in (E) and subjected to MWM test. Latency to escape to a hidden platform (time to find the platform shown in the right panel) during a 5-day training period is shown (*n* = 6 mice in mCherry injection APP/PS1 mice (Ctrl), *n* = 6 mice in Hsc70-WT injection APP/PS1 mice (Hsc70 WT) and *n* = 6 mice in Hsc70-S85A injection APP/PS1 mice (Hsc70 S85A); mean ± SD; *P < 0.05, *t*-test). (G) Mice were treated as in (E) and brain tissues were analyzed for soluble and insoluble Aβ_1-42_ levels, using ELISA (mean ± SD; *P < 0.05, **P < 0.01, *t*-test). (H) Mice were treated as in (E) and immunohistochemistry of the hippocampus was performed to stain for amyloid plaques (6E10 antibody, green) and nuclei (DAPI, blue). Scale bar, 1000 μm. (I) Mice were treated as in (E) and mouse brain tissue lysates were immunoblotted with indicated antibodies (each band represents random independent mouse sample, *n* = 3 mice in WT, *n* = 3 mice in mCherry injection APP/PS1 mice (Ctrl), *n* = 3 mice in Hsc70-WT injection APP/PS1 mice (Hsc70 WT), *n* = 3 mice in Hsc70-S85A injection APP/PS1 mice (Hsc70 S85A)), the numbers under the blots represent the grayscale quantification normalized to tubulin.

APP/PS1 mice develop AD-like cognitive deficits that can be assessed using Morris water maze test (Keowkase 2010). To investigate the effect of Metformin-induced CMA activation on memory loss and AD-associated molecular pathogenesis markers, the mice were treated with water (Veh) or Metformin (Met) in drinking water for 12 weeks. Notably, Metformin-treated APP/PS1 mice showed improved learning and spatial memory (Fig. 6A), displayed strongly reduced levels of insoluble Aβ_1-42_ in the whole brain (Fig. 6B), and a decrease in the Aβ plaque levels in the hippocampus (Fig. 6C). Moreover, the activation of astrocytes in the hippocampus, as judged by GFAP staining, was also reduced upon following Metformin treatment (Fig. 6C). Importantly, Metformin treatment also significantly reduced the protein levels of APP and induced Hsc70 phosphorylation at Ser85, consistent with our findings in cell culture (Fig. 6D).

In order to test whether activation of CMA via Hsc70 overexpression can also alleviate the APP/PS1 phenotypes, we overexpressed Hsc70-WT and Hsc70-S85A in the hippocampus of the mice (Fig. 6E). AAV particles expressing either control vector (mCherry), Hsc70-WT, or Hsc70-S85A were injected into the hippocampus of 16-weeks-old APP/PS1 mice, and 12 weeks later, their behavioral and molecular phenotypes were examined. Using the Morris water maze test, we found that Hsc70-WT overexpression alleviated the cognitive deficits of APP/PS1 mice, and the S85A mutation partially reduced that effect (Fig. 6F). Similarly, insoluble levels of Aβ_1-42_ were decreased in the whole brain tissues of APP/PS1 mice upon overexpression Hsc70-WT, but not Hsc70-S85A (Fig. 6G). Moreover, Aβ plaque levels (Fig. 6H) and APP protein levels (Fig. 6I) were significantly reduced upon overexpression of Hsc70-WT, but not Hsc70-S85A, in the hippocampus of the APP/PS1 mice.

In summary, our results show that CMA activation by either Metformin treatment or overexpression of Hsc70 can decrease the insoluble Aβ_1-42_ burden, Aβ plaque levels, induce degradation of APP and alleviate cognitive deficits of the APP/PS1 model of Alzheimer’s disease.

## Discussion

Our study identifies Metformin as a novel CMA activator, sheds light on the mechanism of CMA activation by Metformin, and identifies APP as a novel CMA substrate, suggesting that Metformin-induced CMA activation and degradation of APP and Aβ can have beneficial effects in the context of pathogenesis of the Alzheimer’s disease.

We identify the TAK1-IKKα/β signaling to be the first regulatory input into the CMA pathway and show that IKKα/β-mediated phosphorylation of Hsc70 at Ser85 controls its ability to interact with its substrates. Our study, thus, places drugs that target TAK1 and IKKα/β as novel inhibitors of CMA. Future studies will determine how the TAK1-IKKα/β signaling, activated by physiological agonists such as TNFα (Wang 2001; Ea et al. 2006), controls CMA in Metformin-unrelated contexts.

Hsc70 is a constitutively-expressed protein and plays an important role in maintain and regulating cellular functions (Liu et al. 2012; Stricher et al. 2013). The structure of Hsc70 includes three parts: a highly conservative amino-terminal adenosine triphosphatase (ATPase) domain (residues 1–384), a peptide (substrate) binding domain (residues 385–543), and a carboxyl-terminal domain (residues 544–646) (Smith et al. 1998; Stricher et al. 2013). Our study find that Metformin treatment enhanced the phosphorylation of Hsc70 on Ser85 which located in ATPase domain of Hsc70. Previously studies clarified that ATP hydrolysis by Hsc70 induces a global conformational change and further causes substrate binding by Hsc70 and there are some co-factors like JDPs or Hsp150alpha that associate ATP consumption with conformational change of Hsc70, thus affecting its binding to the substrates (Yamagishi et al. 2004; Wentink 2020). We speculate that the interaction of IKKα/β with Hsc70 can stimulate the ATPase activity, changes its conformation and enhances its affinity with substrates. Ser85 plays an important role in this process, when Ser85 was mutated, ATPase activity is inhibited and the conformational change was blocked which weakens the binding with substrates. This is in accordance with our results which S85A mutation blocked the interaction of Hsc70 and substrate proteins HK2 and PKM2.

Under physiological conditions, IKKα and IKKβ have been reported to exist as a heterodimer (Mercurio 1997; Woronicz et al. 1997). Our results showed that CMA activation by Metformin triggers the activation of IKK activity and phosphorylation of Hsc70 at Ser85, in an IKK-dependent manner. We find that knockdown of either IKKα or IKKβ rescues the degradation of CMA substrates, suggesting that IKKα/β heterodimer is required for Metformin-induced activation of CMA. This is in accordance with our *in vitro* kinase assays showing that the combination of IKKα and IKKβ further increases the phosphorylation of Hsc70 at Ser85 (Fig. 4B) and previous reports of the IKK complex activation during starvation-induced autophagy (Criollo 2010; Comb et al. 2012).

We found that Metformin-induced CMA activation is dependent on TAK1, expression of which has been shown to decline with age and linked to neurodegenerative diseases such as AD and Parkinson’s disease (Xu 2018). Whether age-dependent loss of TAK1 and, based on the findings of our study, presumably, a decrease of the TAK1-IKKα/β-Hsc70 signaling that would result in a decrease of CMA-mediated clearance of APP contributes to the pathogenesis of AD or other neurodegenerative diseases remains to be investigated.

The mean plasma concentrations of Metformin in humans are reported to be around 20 μmol/L (Graham 2011). Metformin can rapidly cross the blood brain barrier (BBB) once in circulation and distributes well into several brain regions in Wistar rats and the drug concentration in brain tissue was almost equal to the plasma concentration (Labuzek 2010). In this study, we discover that APP and its AD-associated mutant are novel CMA substrates. We find that APP is degraded in an IKKα/β-dependent manner following Metformin treatment, both in SH-SY5Y cells and in APP/PS1 mice, and that phosphorylation of Hsc70 by IKKα/β promotes its interaction with APP and is critical for the alleviation of *in vitro* APP-induced cytotoxicity and *in vivo* rescue of both behavioral and molecular AD phenotypes. These findings suggest that low IKKα/β activity may contribute to diminished clearance of APP and potentially link IKKα/β kinases to the molecular mechanism of AD pathogenesis. Future studies will shed light on whether TAK1 or IKKα/β inhibitors can exacerbate AD phenotypes in APP/PS1 mice and whether pro-inflammatory pathways upstream of TAK1-IKKα/β signaling play a role in the pathogenesis of AD.

Upregulation of APP and compromised CMA are common pathological features that contribute to the development of AD both in transgenic animal models and in human neurons affected in AD (Wang 2017; Kaushik and Cuervo 2018). Our study opens new avenues for concurrent CMA activation and degradation of APP via treatment with Metformin or overexpression of Hsc70. We demonstrate that both Metformin-induced activation of CMA and activation of CMA by overexpression of Hsc70 in the hippocampus of APP/PS1 mice can alleviate the cognitive deficits seen in this mouse model of AD, indicating that CMA activation can indeed be a viable therapeutic avenue for treatment of the AD.

Some clinical studies show that long-term Metformin treatment has been shown to decrease the risk of cognitive decline (Ng 2014). Metformin has also been shown to reduce abnormal accumulation of Aβ and to improve memory (Chen 2016). On the basis of reviewing scientific literature we can imply that there is a tendency towards the application of Metformin in the treatment of AD. However, the exact mechanism of Metformin’s advantageous activity in AD is not fully understood. Metformin was suggested to be associated with the reduction of β-secretase, the rate-limiting enzyme responsible for the conversion of APP to Aβ (Hettich 2014). Our results delineate a molecular mechanism of Metformin-induced CMA and degradation of APP and Aβ, thus, suggest an additional molecular pathway through which Metformin may alleviate the progression of AD. More broadly, our study raises the possibility that activation of CMA by Metformin could also be beneficial to other CMA-related diseases, as well as other aging-associated and aggregation-associated pathologies, including various neurodegenerative diseases.

CMA activation selectively degrades the proteome (Kirchner 2019) and therefore is expected to differentially affect various signaling networks. How Metformin-induced CMA activation affects various signaling networks in the context of, for example, pathways related to TNFα-induced inflammation, and whether this plays a role in the mechanism of action of Metformin in the context of type 2 diabetes, or the reported effects of Metformin on cancers (Evans et al. 2005) and AD (Campbell 2018), remains to be elucidated.

## Materials and methods

### Cell culture

HEK293T, SH-SY5Y, MEF AMPK WT, MEF AMPK DKO, HEK293 ATG5 WT, HEK293 ATG5 KO, Hela ATG8 WT, Hela ATG8 KO and PC12 cells were grown in DMEM medium (Hyclone, with L-glutamine, with 4.5 g/L glucose, without pyruvate); H4 cells were grown in DMEM medium (Hyclone, with L-glutamine, with 4.5 g/L glucose, with pyruvate). These media were supplemented with 10% FBS (Gibco^TM^), 1% Penicillin/Streptomycin (Gibco^TM^). Doxycycline-inducible 293THK stable cell lines were generated by co-transfecting HP138-HK2 and HP216 plasmids (a gift from Dr. Hui Yang) into HEK293T cells using Lipofectamine ^TM^ 2000 (Invitrogen^TM^) and selected with 10 µg/mL puromycin (Sangon® Biotech), obtained monoclonal cell lines by flow cytometry.

### High throughput screening

FDA-approved drugs or drug candidates library were purchased from Topscience, Inc. (Shanghai, China). Drug concentration in the high-throughput screen was 10 μg/mL. Spautin-1 (10 μmol/L) and AC220 (2 μmol/L) treatment was used as a positive control to induce Chaperone mediated autophagy (CMA), DMSO was used as a negative control since compounds from the screen library were dissolved in DMSO. The inducers of CMA were screened using the 293THK stable cell line. The high-throughput screening was done using flow cytometry. Every plate has negative control (DMSO) and positive control (Spautin-1 + AC220), every plate has three replicates, and the screen Z-factor of every plate was > 0.5. There were two high-throughput screenings. In the first screening, 293THK cells were pretreated with or without DOX and were plated in 96-well plates at a density of 2 × 10^4^ cells per well, treated with FDA-approved drugs or drug candidates for 24 h, the fluorescence of HK2-GFP was analyzed by flow cytometry. In the second screening, 293THK cells were pretreated with or without DOX and were plated in 96-well plates at a density of 2 × 10^4^ cells per well, transfected with siRNA of Lamp2a for 48 h, then treated with the 195 hits from the primary high-throughput screening for another 12 h, the HK2-GFP fluorescence was analyzed by flow cytometry.

### Reagents and antibody generation

The chemicals and their sources are as follows: Metformin (#A506198), DOX (#A603456), Puromycin (#A606719), NH_4_Cl (#A501569) from Sangon® Biotech; TPCA1 (#S2824), MG132 (#S2619), Bafilomycin A1 (#S1413), E-64D (#S7393), Leupeptin hemisulfate (#S7380), Spautin-1 (#S7888), AC220 (#S1526) from SelleckChem; Aβ_25-35_ (#A4559) from Sigma. Anti-Flag (DYKDDDDK) Affinity Gel (#B23102), Anti-HA magnetic beads (#B26202) and Anti-Myc magnetic beads (#B26301) were purchased from Bimake. Pierce™ Protein A/G Magnetic Agarose Beads (#78610) were purchased from Thermo Fisher Scientific. Lipofectamine ^TM^ 2000 (#1901433) and Lipofectamine ^TM^ 3000 (#2067450) were from Invitrogen. The following antibodies were used in this study: HK2 (#22029-1-AP), APP (#60342) from ProteinTech ^TM^; GFP (B-2) (#sc-9996), Hsc70 (B-6) (#sc-7298) from Santa Cruz; β-tubulin (#M1305-2), Flag-Tag (#M1403-2), HA-Tag (#0906-1), Myc-Tag (#R1208-1) from HuaAn Biotechnology; Lamp2a (#ab18528) from Abcam; PKM2 (#4053), phospho-AMPKα (Thr172) (#2535), phospho-IKKα/β (ser176/180) (#2694), AMPKα (#5832), IKKα (#11930), IKKβ (#8943), APP/β-Amyloid (#2450), TAK1 (#5206), ATG5 (#12994S) from Cell Signaling Technology®. LC3 (#L8918) from Sigma. The anti-phospho-human Hsc70 ser85 antibody was raised against the region near Ser85 phosphorylation site of Hsc70. The phosphorylated synthetic peptide [DAVVQSDMKHWPFMC] was used for immunization in the rabbits. The antibody was generated by GenScript (Nanjing China). The secondary antibodies for Western blot were used: Goat anti-Mouse IgG (H + L) (#31430, Thermo Fisher Scientific), Goat anti-Rabbit IgG (H + L) (#31460, Thermo Fisher Scientific).

### Molecular cloning and siRNA knockdown

Hsc70-Flag, Lamp2a-Flag, λ-phosphatase-Flag, IKKα-Myc, IKKβ-HA, APP, were cloned into pcDNA5 via *Bam*HI and *Ava*I restriction enzymes by PCR amplifying the ORFs from cDNA templates of *Hsc70* (#19514, Addgene), *Lamp2a* (#86146, Addgene), *APP* (#114193, Addgene), λ-phosphatase, *IKKα* and *IKKβ* were presented by Life Sciences Institute, Zhejiang University. Molecular cloning was performed using T4 DNA ligase and transformed into DH5α *E*.* coli* cells. Plasmid purifications and extractions were performed using the NucleoBond Xtra Midi kit (Macherey-Nagel). siRNA sequences were detailed in Table S1, and siRNA were transformed into HEK293T, H4, SH-SY5Y, 293THK, PC12 cells with Lipofectamine ^TM^ 3000 (Invitrogen), according to the manufacturer’s protocol.

### Flow cytometry

The fluorescence of HK2-GFP was analyzed by flow cytometry. 293THK cells were pretreated with or without 1 μg/mL DOX and treated with indicated methods. The cells were analyzed by flow cytometry (Beckman Coulter Cytoflex), and data were analyzed by CytExpert2.3, the FlowJo software. 293THK without DOX was used as a negative control to distinguish between negative and positive fluorescence cells.

### Protein purification and *in vitro* kinase assays

For purification of IKKα-Myc and IKKβ-HA, pcDNA5-IKKα-Myc and pcDNA5-IKKβ-HA plasmids were transfected into HEK293T cells for 24 h. Cells were lysed in 1 mL of lysis buffer (TAP) (20 mmol/L Tris-HCl (pH 7.5), 150 mmol/L NaCl, 0.5% NP-40, 1 mmol/L NaF, 1 mmol/L Na_3_VO_4_, 1 mmol/L EDTA, Protease inhibitor cocktail (Bimake, added fresh)), and incubated with anti-Myc magnetic beads and anti-HA magnetic beads (after washing the beads with PBS, twice) for 6 h on a rotating wheel at 4 °C. The beads were washed three times with TAP buffer and treated with CIP (#M0290, Biolabs) for 30 min at 37 °C. The kinase assays were performed with recombinant Human Hsc70 proteins (#ab78431, Abcam). The proteins were incubated in 30 μL of kinase buffer (25 mmol/L Tris-HCl (pH 7.5), 5 mmol/L β-glycerophosphate, 2 mmol/L dithiothreitol (DTT), 0.1 mmol/L Na_3_VO_4_, 10 mmol/L MgCl_2_) supplemented with phosphatase inhibitor cocktail (#b15001, Bimake), with or without 100 μmol/L ATP for 30 min at 30 °C. The reactions were stopped by adding SDS-PAGE 2× loading buffer (100 mmol/L Tris-HCl (pH 6.8), 4% SDS, 20% glycerol, 0.2 mol/L DTT, 0.1% bromophenol blue) and heating at 100 °C for 10 min.

### Purification of TAK1

For purification of TAK1-Flag, pcDNA5-TAK1-Flag were transfected into HEK293T cells for 24 h. Cells were lysed in 1 mL of lysis buffer (TAP) (20 mmol/L Tris-HCl (pH 7.5), 150 mmol/L NaCl, 0.5% NP-40, 1 mmol/L NaF, 1 mmol/L Na_3_VO_4_, 1 mmol/L EDTA, Protease inhibitor cocktail (Bimake, added fresh)), and incubated with anti-Flag magnetic beads (after washing the beads with PBS, twice) for 6 h on a rotating wheel at 4 °C. TAK1-Flag were eluted using 3× Flag peptide. The protein was dissolved into TBS and diluted to a final concentration of 50 μg/mL before the protein thermal shift assay.

### Protein thermal shift assay

The protein thermal shift assay was performed as described previously (Groftehauge et al. 2015; Xu 2020). To determine stability, purified TAK1 were added to SYPRO Orange dye. Metformin and 5z-7-oxozeaenol were added to the proteins to make a final concentration of 20 mmol/L and 100 μmol/L and incubated at 4 °C for 1 h. The experiments were performed in 96-well plates specific for the real-time PCR instrument with a total volume of 20 μL/well. The assay plate was placed into the ABI-7500 Fast Real-time PCR system. The reaction was run from 25 °C, ramping up in increments of 0.05 °C/s to a final temperature of 99 °C with fluorescence detection throughout the experiment to generate a dataset. The melting temperatures of the protein was determined by performing nonlinear fitting of the data set to a Boltzmann sigmoidal curve in GraphPad Prism.

### Immunoprecipitation

For purification of Hsc70-WT-Flag, Hsc70-S85A-Flag, Lamp2a-Flag, APP-WT, and APP-M7, pcDNA5-Hsc70-WT-Flag, pcDNA5-Hsc70-S85A-Flag, pcDNA5-Lamp2a-Flag, pcDNA5-APP, and pcDNA5-APP-M7 plasmids were transfected into HEK293T cells or SH-SY5Y cells for 24 h. Cells were washed with PBS and lysed in 1 mL of lysis buffer (TAP) (20 mmol/L Tris-HCl (pH 7.5), 150 mmol/L NaCl, 0.5% NP-40, 1 mmol/L NaF, 1 mmol/L Na_3_VO_4_, 1 mmol/L EDTA, Protease inhibitor cocktail (Bimake, add fresh) for 30 min, incubated with anti-Flag (DYKDDDDK) beads or APP antibodies incubated with Pierce™ Protein A/G Magnetic Agarose Beads (#78610, Thermo Fisher Scientific) for 6 h on a rotating wheel at 4 °C. The beads were washed with TAP three times, 5 min each wash, and SDS-PAGE 2× loading buffer was added, followed by heating at 100 °C for 10 min.

### Immunoblotting

Cell lysates or pull-down samples were added SDS-PAGE 2× loading buffer and heated at 100 °C for 10 min, subjected to 10%–12% SDS-PAGE, and then transferred onto PVDF membranes for 1 h at 0.2 A with semi-dry transfer system of BioRad. Membranes were blocked in PBST buffer containing 5% (*w*/*v*) skimmed milk for 1 h and probed with the indicated antibodies in PBST containing 5% (*w*/*v*) BSA at 4 °C overnight. Detection was performed using HRP-conjugated secondary antibodies and chemiluminescence reagents (#4AW001-500, 4A Biotech Co.). We used ImageJ 1.53a for the analysis of grayscale quantification. The numbers under the blots represent the average value (the ratio to Tubulin) of grayscale quantification.

### Cell death and survival assays

SH-SY5Y cells were transfected with pcDNA5-APP or pcDNA5-APP-M7, co-transfected with or without pcDNA5-Hsc70-WT-Flag, or pcDNA5-Hsc70-S85A-Flag for 24 h using Lipofectamine ^TM^ 3000 (Invitrogen ^TM^), treated with or without Metformin (20 μmol/L) for another 48 h. PC12 cells were transfected with siRNA of Hsc70, Lamp2a and ATG5, treated with or without 20 μmol/L Aβ_25-35,_ and Metformin (20 μmol/L) for 96 h. PC12 cells were treated with Aβ_25-35_ (20 μmol/L) and Metformin (20 μmol/L) for 72 h, with or without TPCA1 (5 μmol/L) for another 24 h. Cell viability was detected using CellTiter-Glo® Luminescent Cell Viability Assay (#G7573 Promega) according to the manufacturer’s instructions.

### Mass spectrometry and data analysis

Hsc70 was trypsin-digested on beads following immunoprecipitation. The resulting peptides were subjected to the phosphopeptide enrichment using TiO_2_ beads. The enriched phospho-peptides were analyzed on the Q Exactive™ HF mass spectrometer (Thermo Fisher Scientific). The identification and quantification of phosphorylated peptides were done using MaxQuant (Cox and Mann 2008). The tandem mass spectra were searched against UniProt human protein database together with a set of commonly observed contaminants. The precursor mass tolerance was set as 20 ppm, and the fragment mass tolerance was set as 0.1 Da. The cysteine carbamidomethylation was set as a static modification, and the methionine oxidation, as well as serine, threonine, and tyrosine phosphorylation, were set as variable modifications. The FDR at peptide spectrum match level was controlled below 1%.

### Proximity ligation assays (PLA)

PLA assay were performed according to Duolink (#DUO92010, Sigma). H4 cells or HEK293T cells were cultured on glass slides, treated with Metformin or transfected with indicated plasmids for the indicated times, washed twice with PBS, fixed with 4% paraformaldehyde for 20 min. Following blocking with 5% FBS supplement with 0.1% Triton X-100 to increase the permeabilization for 1 h, the primary antibodies (Hsc70, PKM2, Lamp2a, Flag) were incubated with the slides overnight at 4 °C. After incubating with the secondary antibodies conjugated with the PLA probes, the signals were amplified through ligation and amplification. The fluorescence analysis was done using Cytation® 3.

### Animal work

Male transgenic APP/PS1 (C57BL/6) mice at the age of 14–16 weeks were purchased from the Model Animal Research Center of Nanjing University (Nanjing, Jiangsu, China) and housed in a pathogen-free environment of experimental animal center in Zhejiang University. All animal studies and experimental procedures were approved by the Animal Care and Use Committee of the animal facility at Zhejiang University. Mice were divided into three groups: normal C57 group (WT, *n* = 7), control APP/PS1 group (APP/PS1 + Veh, *n* = 7), and APP/PS1 treated with Metformin group (APP/PS1 + Met, *n* = 7). The mice in the Metformin group had access to tap water with Metformin freely for 12 weeks.

For Hsc70 overexpression mice, 16 weeks old mice were divided into three groups: APP/PS1 mice injected AAV control (mCherry) group (Ctrl, *n* = 6), APP/PS1 mice injected AAV-Hsc70- WT-Flag-P2A-mCherry group (Hsc70 WT, *n* = 6), APP/PS1 mice injected AAV-Hsc70-S85A-Flag-P2A-mCherry group (Hsc70 S85A, *n* = 6). The AAV encoded mRNA of Hsc70 WT-Flag-P2A-mCherry, Hsc70 S85A-Flag-P2A-mCherry, and mCherry under the control of CAG promoter and packaged into an adeno-associated virus (AAV8) by Vigene biosciences, China. The stereotactic injection was performed in anesthetized mice, and AAV encoding mCherry, Hsc70 WT-Flag-P2A-mCherry or Hsc70 S85A-Flag-P2A-mCherry were injected into the hippocampus (1.5 μL bilateral) of APP/PS1 mice (A/P − 2 mm, M/L − 1.5 mm, D/V − 1.5 mm), and flow rates are 0.3 μL/min, as described previously (Wegmann et al. 2017), under standard aseptic surgery conditions. After finishing the injection, the needle was left in place for 2 min to allow the diffusion of the injected AAV solution. Close the skin incision using glue and let the mouse recover from anesthesia. Injected mice were housed under the standard condition for 12 weeks.

### Morris water maze

The water maze behavioral tests were performed as described previously (Vorhees and Williams 2006). The device is a circular pool (140 cm diameter) filled with water supplemented with titanium dioxide and maintained at 22 °C. A 12 cm diameter platform was placed 1 cm below the water surface in a fixed position. Mice were trained in four quadrants every day for five consecutive days. Each quadrant trial lasted 60 s or until the mouse found the platform. If the mouse did not find the platform within the prescribed time, the experimenter needed to guide the mouse to stand on the platform for 20 s. All parameters were recorded by a video tracking system.

### Immunohistochemistry

The brain samples were collected from the mice, hemisected, and fixed in 4% paraformaldehyde for 24 h. The following procedures were done by HUABIO, China. Tissues were stained with polyclonal rabbit anti-GFAP antibody (#Z033401-2, DAKO, 1:100) to detect astrocytes, mouse anti-β-Amyloid, 1-16 antibody (clone 6E10, #803002, Biolegend®, 1:100) to detect Aβ plaques, followed by secondary antibody staining and imaging with Cytation® 3.

### ELISA for Aβ_1-42_

Mouse brain tissues were prepared according to the brain tissue homogenate protocol described in the ELISA technical guide from Life technologies. The accumulation of Aβ_1-42_ was quantified by ELISA (# KHB3441, Thermo Fisher Scientific).

### Software

The prediction of Hsc70 kinases was made using GPS3.0 software (Xue 2008) using the “Medium” threshold setting. APP amino acid sequences were obtained from NCBI and aligned by Clustal W. The alignment images were obtained using the ESPript 3.0 software. ImageJ 1.53a was used for grayscale quantification of immunoblotting.

### Quantification and statistical analysis

Statistical analyses were performed with GraphPad Prism 7. Data were analyzed with a one-way analysis of variance (ANOVA) test or Student’s *t*-test. Data points are shown as mean ± SD. All experiments contained at least three biological replicates.

## Supplementary Information

Below is the link to the electronic supplementary material.

Supplementary file1 (PDF 1977 KB)

## Data Availability

The datasets generated during and/or analysed during the current study are available from the corresponding author on reasonable request.

## Abbreviations

AAV: Adeno-associated virus
AD: Alzheimer disease
AMPK: AMP activated protein kinase
APP: amyloid precursor protein
Aβ: β-amyloid
CMA: chaperone-mediated autophagy
Hsc70 (HSPA8): heat shock protein family A (Hsp70) member 8
LAMP2A: lysosomal associated membrane protein 2A
Met: Metformin
PKM2: pyruvate kinase isozyme type M2
TAK1: transforming growth factor beta-activated kinase1
