# Correction to: Metformin activates chaperone-mediated autophagy and improves disease pathologies in an Alzheimer disease mouse model

**DOI:** 10.1007/s13238-021-00873-4

**Published:** 2021-11-01

**Authors:** Xiaoyan Xu, Yaqin Sun, Xufeng Cen, Bing Shan, Qingwei Zhao, Tingxue Xie, Zhe Wang, Tingjun Hou, Yu Xue, Mengmeng Zhang, Di Peng, Qiming Sun, Cong Yi, Ayaz Najafov, Hongguang Xia

**Affiliations:** 1 Department of Biochemistry & Research Center of Clinical Pharmacy of The First Affiliated Hospital, Zhejiang University School of Medicine, Hangzhou, 310058 China; 2 Liangzhu Laboratory, Zhejiang University Medical Center, Hangzhou, 311121 China; 3 Department of Cell Biology, Harvard Medical School, Boston, MA 02115 USA; 4 Interdisciplinary Research Center on Biology and Chemistry, Shanghai Institute of Organic Chemistry, Chinese Academy of Sciences, Shanghai, 201203 China; 5 College of Pharmaceutical Sciences, Hangzhou Institute of innovative Medicine, Zhejiang University, Hangzhou, 310058 China; 6 Key Laboratory of Molecular Biophysics of Ministry of Education, Hubei Bioinformatics and Molecular Imaging Key Laboratory, College of Life Science and Technology, Huazhong University of Science and Technology, Wuhan, 430074 China

## Correction to: Protein Cell 10.1007/s13238-021-00858-3

In legend of figure 1, this sentence “(C) 293THK cells were treated as in a” should be corrected as “(C) 293THK cells were treated as in (A)”.

In legend of figure 2, “E-64D (10 μmol/L)” in description of panel (B) should be removed.

In section “Metformin activates chaperone-mediated autophagy” of RESULTS, E-64D in sentence “Metformin-induced degradation of HK2 and PKM2 was blocked by lysosomal inhibitors (E-64D, Bafilomycin A1 and Leupeptin + NH_4_Cl)” should be removed.

The word “upon” in the sentence “Moreover, the activation of astrocytes in the hippocampus, as judged by GFAP staining, was also reduced upon following Metformin treatment (Fig. 6C)” should be removed.

The correct Fig. 2 is shown below.

**Figure 2:**
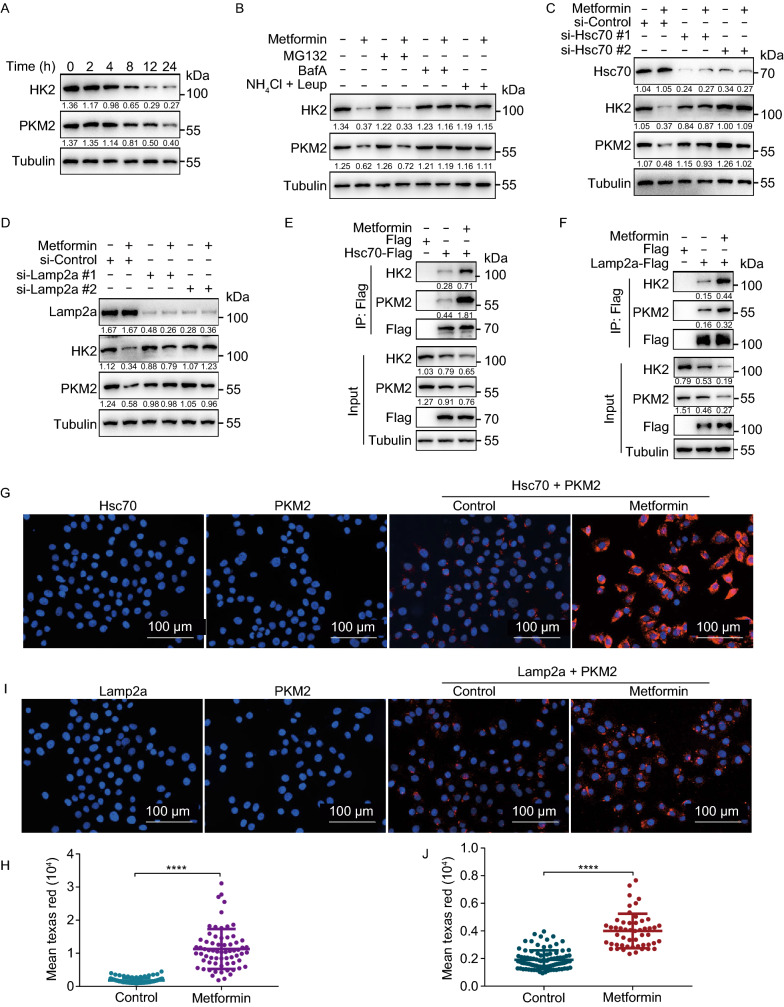
**Metformin activates chaperone-mediated autophagy.** (A) H4 cells were treated with 20 mmol/L Metformin for 2, 4, 8, 12, and 24 h. Cell lysates were immunoblotted with indicated antibodies. (B) H4 cells were treated with 20 mmol/L Metformin with or without MG132 (10 μmol/L), Bafilomycin A1 (100 nmol/L), NH_4_Cl (20 mmol/L), Leupeptin (100 nmol/L), for 12 h. Cell lysates were immunoblotted with indicated antibodies. (C and D) H4 cells were transfected with indicated siRNAs (#1 and #2 represent two different sequences) for 48 h, treated with or without 20 mmol/L Metformin for another 12 h. Cell lysates were immunoblotted with indicated antibodies. (E) HEK293T cells were transfected with Hsc70-Flag for 24 h, treated with or without 20 mmol/L Metformin for another 6 h, the interaction between HK2, PKM2, and Hsc70 were analyzed by immunoprecipitation. (F) HEK293T cells were transfected with Lamp2a-Flag for 24 h, treated with or without 20 mmol/L Metformin for another 6 h, the interaction between HK2, PKM2, and Lamp2a were analyzed by immunoprecipitation. (G) H4 cells were treated with or without 20 mmol/L Metformin for 6 h, PLA assay for endogenous Hsc70 and PKM2 was analyzed by fluorescence microscopy. Scale bar, 100 μm. (H) Quantification of the fluorescence intensity of Texas Red from (G) (data represents mean ± SD; ****P < 0.0001, one-way ANOVA). (I) H4 cells were treated with or without 20 mmol/L Metformin for 6 h, PLA assay for endogenous Lamp2a and PKM2 was analyzed by fluorescence microscopy. Scale bar, 100 μm. (J) Quantification of the fluorescence intensity of Texas Red from (I) (data represents mean ± SD; ****P < 0.0001, one-way ANOVA)

