# Supplementary material for: Metformin activates chaperone-mediated autophagy and improves disease pathologies in an Alzheimer disease mouse model
Source: Protein Cell. 2021 Jul 21;12(10):769–87. doi: 10.1007/s13238-021-00858-3 (PMC8464644; doi:10.1007/s13238-021-00858-3)
Supplement: Supplementary file 1 — Supplementary file1 (PDF 1977 KB) [file 13238_2021_858_MOESM1_ESM.pdf]

Supplementary Figure Legends

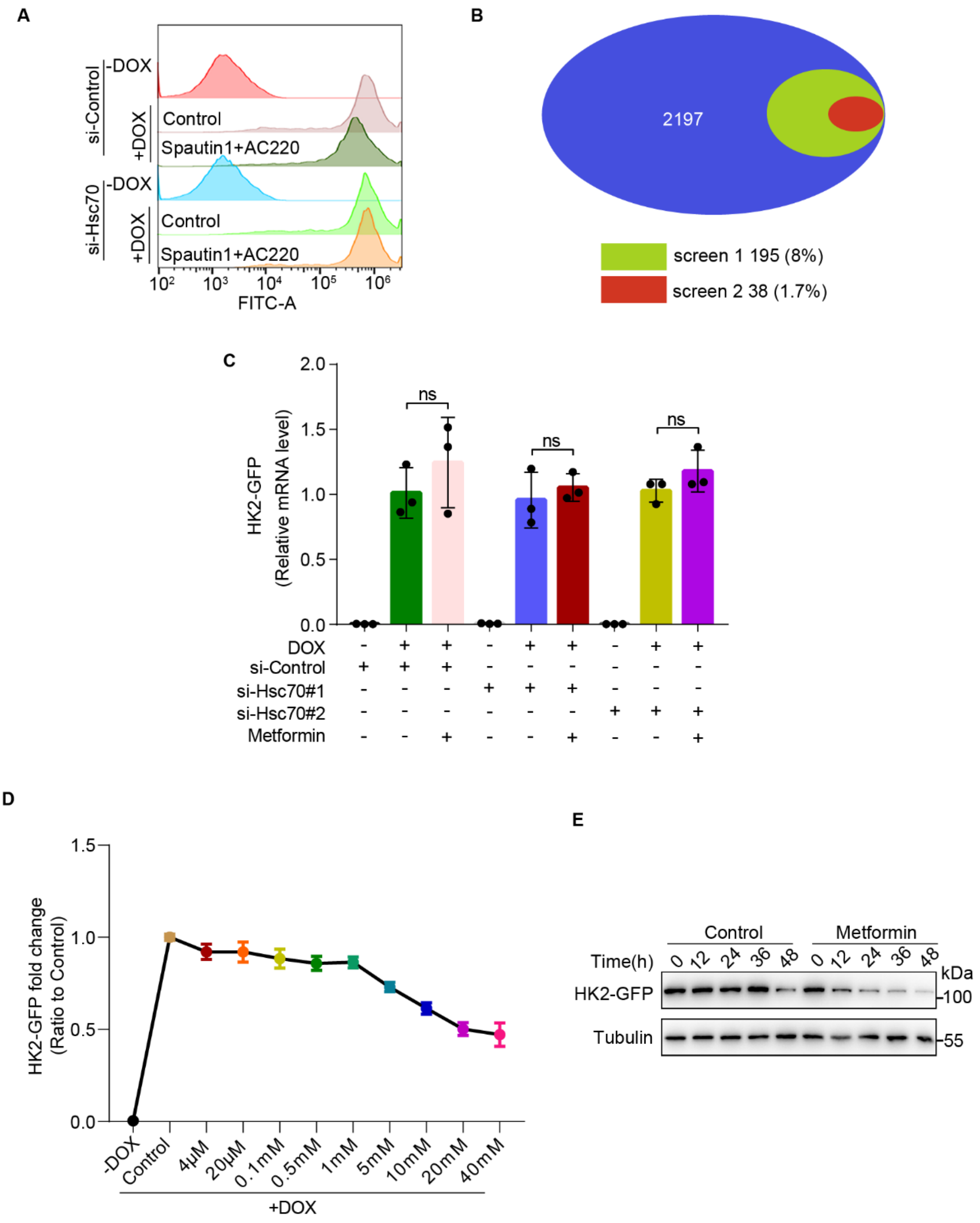

Supplementary Fig. 1. Identification of CMA-inducing drugs by high-throughput screening of FDA-approved drugs.

(**A**) 293THK cells were pretreated with or without 1 μg/mL DOX and treated with Spautin-1 (10 μM) and AC220 (2 μM) for another 12 h, fluorescence of HK2-GFP was analyzed by flow cytometry.

(**B**) 293THK cells were transfected with siRNA of Lamp2a for 48 h, treated with the

selected 195 compounds for another 12 h, the fluorescence of HK2-GFP was analyzed by flow cytometry and compared with scrambled siRNA (si-Control). From here, 38 compounds were isolated.

(**C**) 293THK cells were pretreated with or without 1 μg/mL DOX, transfected with siRNA of Hsc70 for 48 h, treated with or without Metformin for another 12 h, total RNA was extracted by FastPure® Cell/Tissue Total RNA Isolation Kit, mRNA levels of GFP was analyzed by qPCR (data represents Mean ± SD; n = 3, t-test).

(**D**) 293THK cells were pretreated with or without 1 μg/mL DOX and treated with different concentrations of Metformin for 24 h, fluorescence of HK2-GFP was analyzed by flow cytometry.

(**E**) 293THK cells were pretreated with 1 μg/mL DOX, treated with and without Metformin (20 mM) for 0, 12, 24, 36 and 48 h, cell lysates were immunoblotted with indicated antibodies.

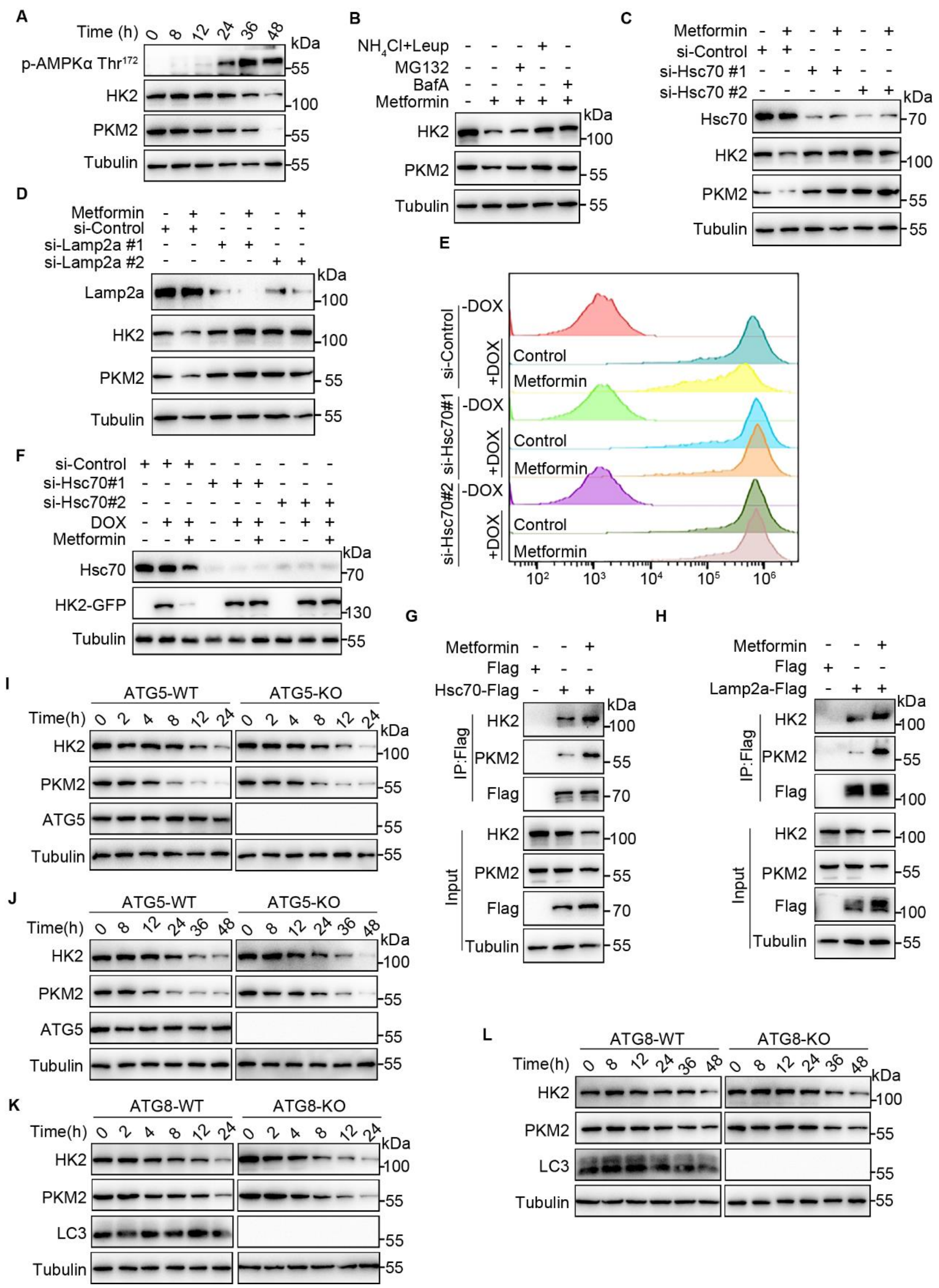

Supplementary Fig. 2. Metformin activates chaperone-mediated autophagy.

(**A**) H4 cells were treated with 20 μM Metformin for 8, 12, 24, 36 and 48 h, cell lysates were immunoblotted with indicated antibodies.

(**B**) H4 cells were pretreated with 20 μM Metformin for 36 h, treated with MG132 (10

μM), Bafilomycin A1 (100 nM), NH_4Cl (20 mM), or Leupeptin (100 nM) for another 12 h. Cell lysates were immunoblotted with indicated antibodies.

(**C, D**) H4 cells were transfected with indicated siRNA for 12 h, treated with or without 20 μM Metformin for another 48 h, cell lysates were immunoblotted with indicated antibodies.

(**E**) 293THK cells were pretreated with or without 1 μg/mL DOX, transfected with siRNA of Hsc70 for 12 h, treated with or without 20 μM Metformin for another 48 h, fluorescence of HK2-GFP was analyzed by flow cytometry.

(**F**) 293THK cells were treated as shown in (E). Cell lysates were immunoblotted with indicate antibodies.

(**G, H**) HEK293T cells were transfected with indicated plasmids for 24 h, treated with or without 20 μM Metformin for 12 h, the interaction of HK2, PKM2 with Hsc70 and Lamp2a were analyzed by immunoprecipitation.

(**I, J**) ATG5 wild type (WT) and ATG5 knockout (KO) HEK293 cells were treated with 20 mM (I) or 20 μM (J) Metformin for indicated time, cell lysates were immunoblotted with indicated antibodies.

(**K, L**) ATG8 wild type (WT) and ATG8 knockout (KO) Hela cells were treated with 20 mM (K) or 20 μM (L) Metformin for indicated time, cell lysates were immunoblotted with indicated antibodies.

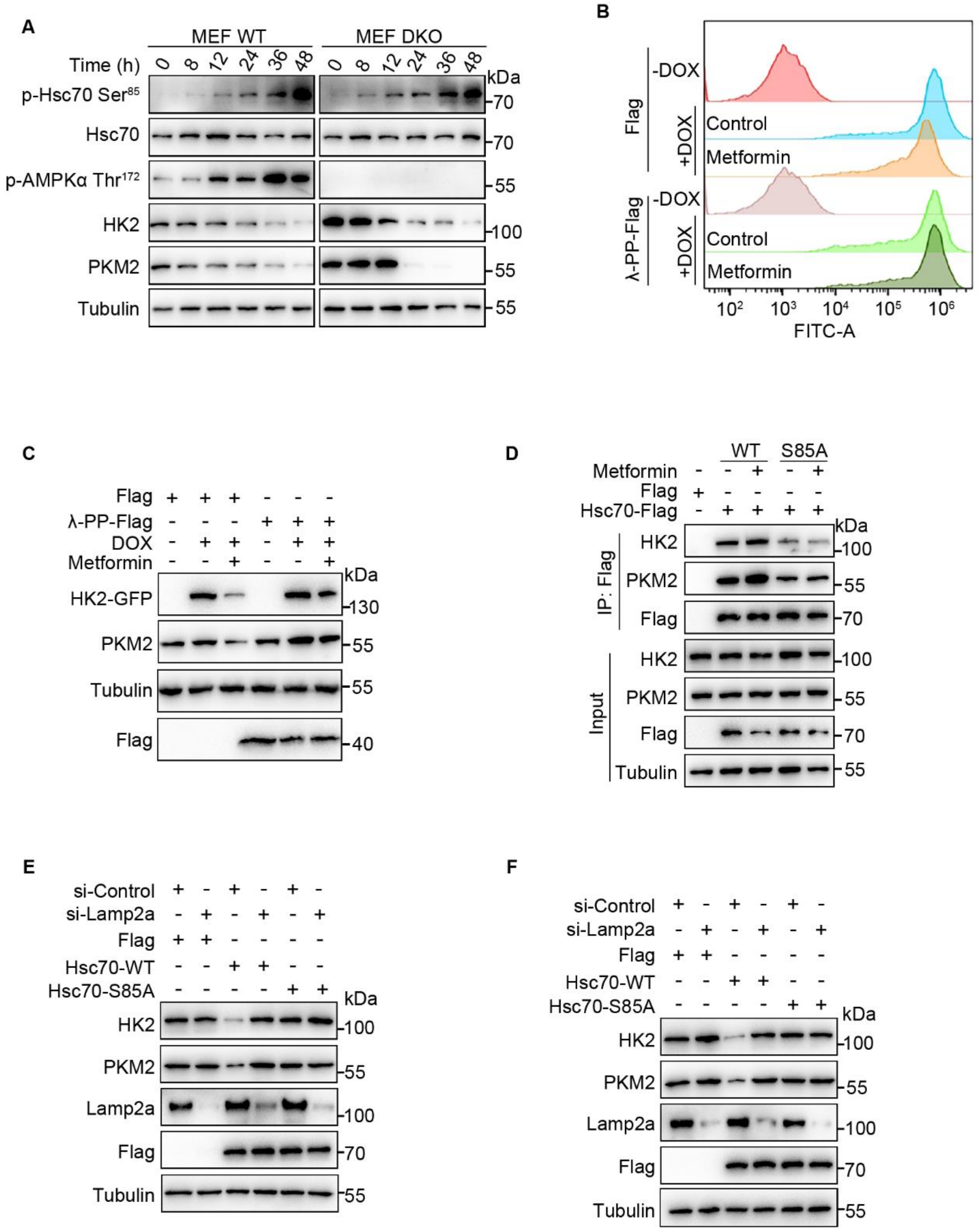

Supplementary Fig. 3. Metformin induces phosphorylation of Hsc70 at Ser85 in an AMPK-independent manner.

(**A**) AMPK wild type (WT) and α1/α2 double knockout (DKO) MEF cells were incubated with 20 μM Metformin for 8, 12, 24, 36 and 48 h, cell lysates were immunoblotted with indicated antibodies.

(**B**) 293THK cells were pretreated with or without 1 μg/mL DOX and transfected with indicated plasmids for 12 h, treated with 20 μM Metformin for another 48 h, fluorescence of HK2-GFP was analyzed by flow cytometry.

(**C**) 293THK cells were treated as shown in (B). Cell lysates were immunoblotted with indicated antibodies.

(**D**) HEK293T cells were transfected with Hsc70 WT-Flag or Hsc70 S85A-Flag for 24 h, treated with or without 20 μM Metformin for another 12 h. The interaction of Hsc70 with HK2 and PKM2 was analyzed by immunoprecipitation.

(**E, F**) H4 (E) or HEK293T (F) cells were transfected with siRNA of Lamp2a for 36 h, then transfected with Hsc70-WT, Hsc70-S85A for another 24 h, cell lysates were immunoblotted with indicated antibodies.

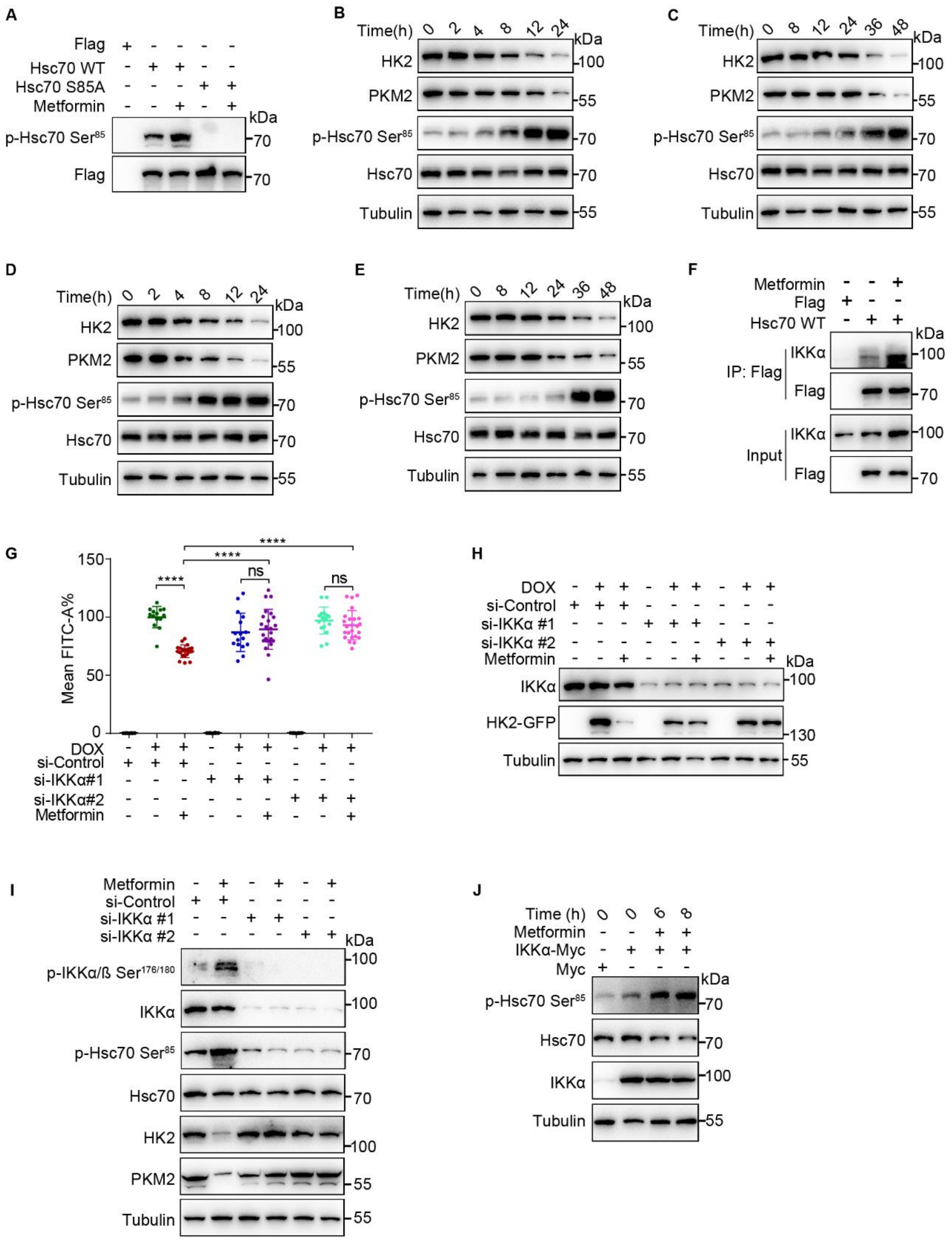

Supplementary Fig. 4 Metformin induces phosphorylation of Hsc70 at Ser85 via IKKα/β activation.

(**A**) HEK293T cells were transfected with Hsc70 WT-Flag or Hsc70 S85A-Flag for 24 h, treated with or without Metformin (20 mM) for another 8 h, Hsc70 was immunoprecipitated with Flag beads and immunoblotted with anti-Flag and anti-p-Hsc70 Ser^{85} antibodies.

(**B, C**) H4 cells were treated with 20 mM (B) or 20 μM (C) Metformin for indicated times,

cell lysates were immunoblotted with indicated antibodies.

(**D, E**) HEK293T cells were treated with 20 mM (D) or 20 μM (E) Metformin for indicated time, cell lysates were immunoblotted with indicated antibodies.

(**F**) HEK293T cells were transfected with Flag or Hsc70 WT-Flag for 24 h, treated with or without 20 mM Metformin for another 6 h, the interaction of IKKα and Hsc70 was detected by immunoprecipitation.

(**G**) 293THK cells were pretreated with or without 1 μg/mL DOX and transfected with siRNA of IKKα for 48 h, treated with or without 20 mM Metformin for another 12 h, fluorescence of HK2-GFP was analyzed by flow cytometry (data represents Mean ± SD; ****p < 0.0001, one-way ANOVA).

(**H**) 293THK cells were treated as in (G). Cell lysates were immunoblotted with indicated antibodies.

(**I**) H4 cells were transfected with siRNA of IKKα for 12 h, treated with or without 20 μM Metformin for another 48 h, cell lysates were immunoblotted with indicated antibodies.

(**J**) HEK293T cells were transfected with IKKα-Myc for 24 h, treated with or without 20 mM Metformin for 6 h and 8 h, and cell lysates were immunoblotted with indicated antibodies.

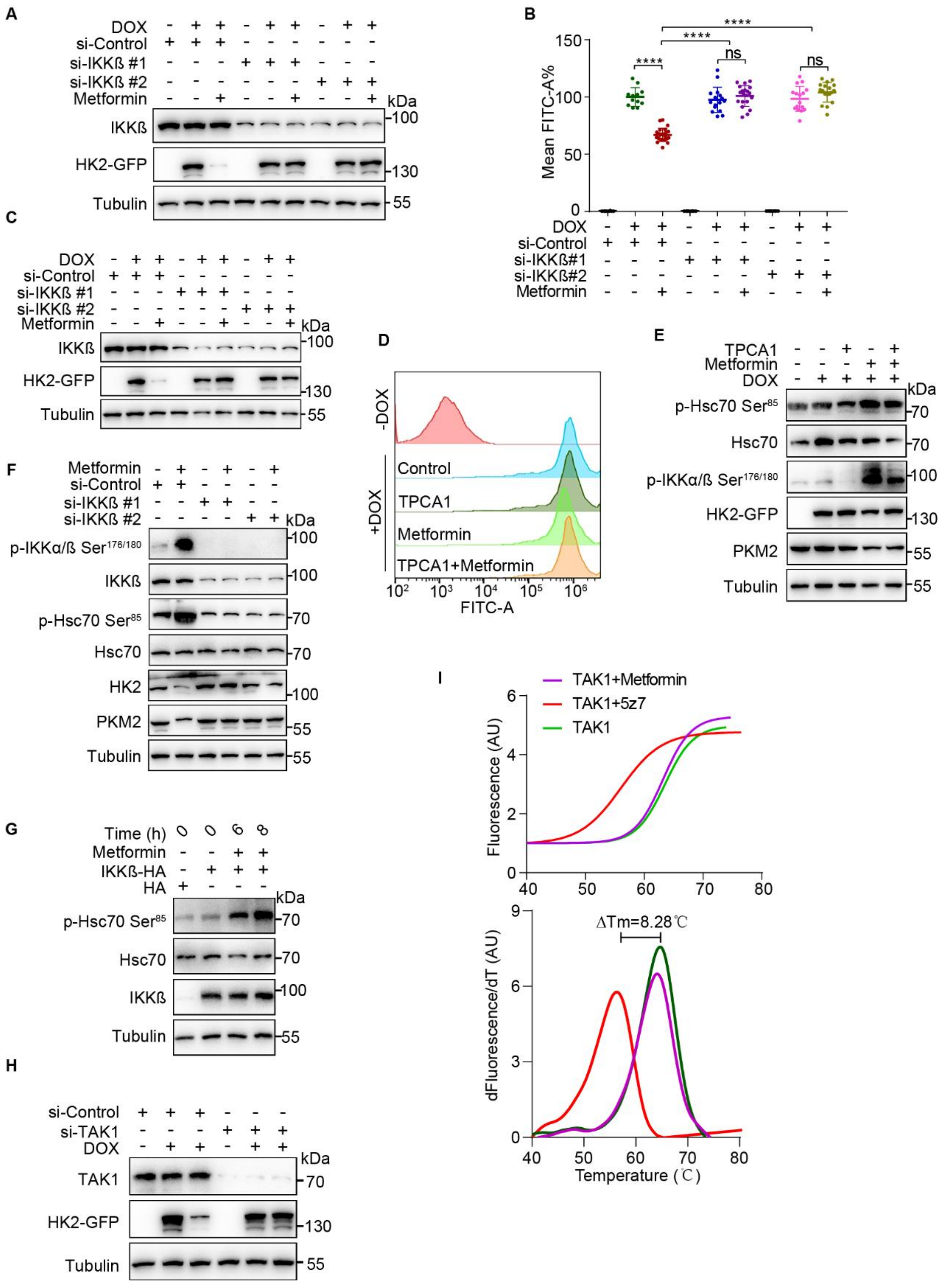

Supplementary Fig. 5 Metformin induces phosphorylation of Hsc70 at Ser85 via IKKα/β activation in a TAK1-dependent manner.

(**A**) 293THK cells were pretreated with or without 1 µg/mL DOX, and transfected with indicated siRNA for 48 h, treated with or without 20 mM Metformin for another 12 h, cell lysates were immunoblotted with indicated antibodies.

(**B**) 293THK cells were pretreated with or without 1 μg/mL DOX and transfected with siRNA of IKKβ for 12 h, treated with or without 20 μM Metformin for another 48 h, fluorescence of HK2-GFP was analyzed by flow cytometry (data represents Mean ± SD; ****$p < 0.0001$, one-way ANOVA).

(**C**) 293THK cell were treated as in (B). Cell lysates were immunoblotted with indicated antibodies.

(**D**) 293THK cells were pretreated with or without 1 μg/mL DOX, treated with 20 μM Metformin for 36 h, and with or without 5 μM TPCA1 for another 12 h, fluorescence of HK2-GFP was analyzed by flow cytometry.

(**E**) 293THK cells were treated as shown in (D). Cell lysates were immunoblotted with indicated antibodies.

(**F**) H4 cells were transfected with siRNA of IKKβ for 12 h, treated with or without 20 μM Metformin for another 48 h, cell lysates were immunoblotted with indicated antibodies.

(**G**) HEK293T cells were transfected with IKKβ for 24 h, treated with or without 20 mM Metformin for 6 h and 8 h. Cell lysates were immunoblotted with indicated antibodies.

(**H**) 293THK cells were pretreated with or without 1 μg/mL DOX and transfected with siRNA of TAK1 for 12 h, treated with or without 20 μM Metformin for 48 h, cell lysates were immunoblotted with indicated antibodies.

(**I**) In vitro binding of Metformin and TAK1 inhibitor 5z-7-oxozeaenol (5z7) to TAK1-Flag as determined by thermal shift assay (#4462263, Thermo Fisher Scientific).

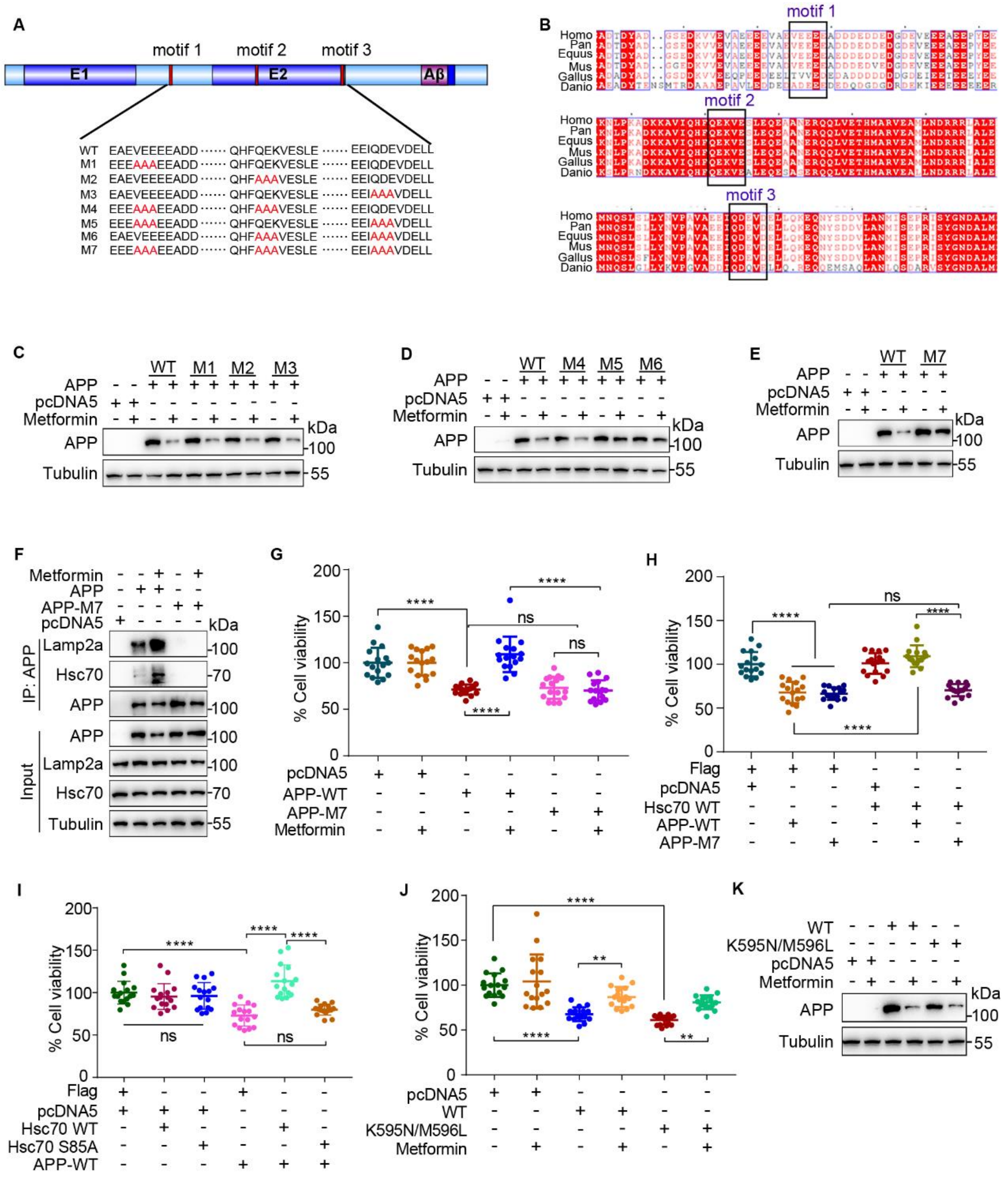

Supplementary Fig. 6 Metformin induces CMA-mediated APP degradation and alleviates the cytotoxic effects of APP.

(**A**) The schematic diagram of APP indicating the position of the three KFERQ-like motifs (red rectangles) and alignments showing the mutations made to inactivate these motifs.

(**B**) The alignment of APP KFERQ-like motifs indicating their conservation across indicated species.

(**C**) SH-SY5Y cells were transfected with APP-WT, APP-M1, APP-M2, APP-M3 for 24 h, treated with Metformin (20 μM) for 48 h. Cell lysates were immunoblotted with anti-

APP and anti-Tubulin antibodies.

(**D**) SH-SY5Y cells were transfected with APP-WT, APP-M4, APP-M5, APP-M6 for 24 h, treated with Metformin (20 μM) for 48 h. Cell lysates were immunoblotted with anti-APP and anti-Tubulin antibodies.

(**E**) SH-SY5Y cells were transfected with APP-WT, APP-M7 for 24 h, treated with Metformin (20 μM) for 48 h. Cell lysates were immunoblotted with anti-APP and anti-Tubulin antibodies.

(**F**) SH-SY5Y cells were transfected with APP-WT and APP-M7 for 24 h, treated with or without Metformin (20 μM) for 12 h, the interaction between APP and Hsc70, Lamp2a were analyzed by immunoprecipitation.

(**G**) SH-SY5Y cells were transfected with APP-WT or APP-M7, treated with or without Metformin (20 μM) for 48 h. Cell viability was determined using CellTiterGlo® assay (data represents Mean ± SD; ****p < 0.0001, one-way ANOVA).

(**H**) SH-SY5Y cells were co-transfected with Hsc70-WT-Flag, APP-WT or APP-M7 for 48 h. Cell viability was determined using CellTiterGlo® assay (data represents Mean ± SD; ****p < 0.0001, one-way ANOVA).

(**I**) SH-SY5Y cells were co-transfected with APP-WT, Hsc70-WT-Flag or Hsc70-S85A-Flag for 48 h. Cell viability was determined using CellTiterGlo® assay (data represents Mean ± SD; ****p < 0.0001, one-way ANOVA).

(**J**) SH-SY5Y cells were transfected with APP-WT or APP K595N/M596L, treated with or without Metformin (20 μM) for 48 h. Cell viability was determined using CellTiterGlo® assay (data represents Mean ± SD; **p < 0.01, ****p < 0.0001, one-way ANOVA).

(**K**) SH-SY5Y cells were transfected with APP-WT or APP K595N/M596L for 24 h, treated with Metformin (20 μM) for another 48 h. Cell lysates were immunoblotted with anti-APP and anti-Tubulin antibodies.

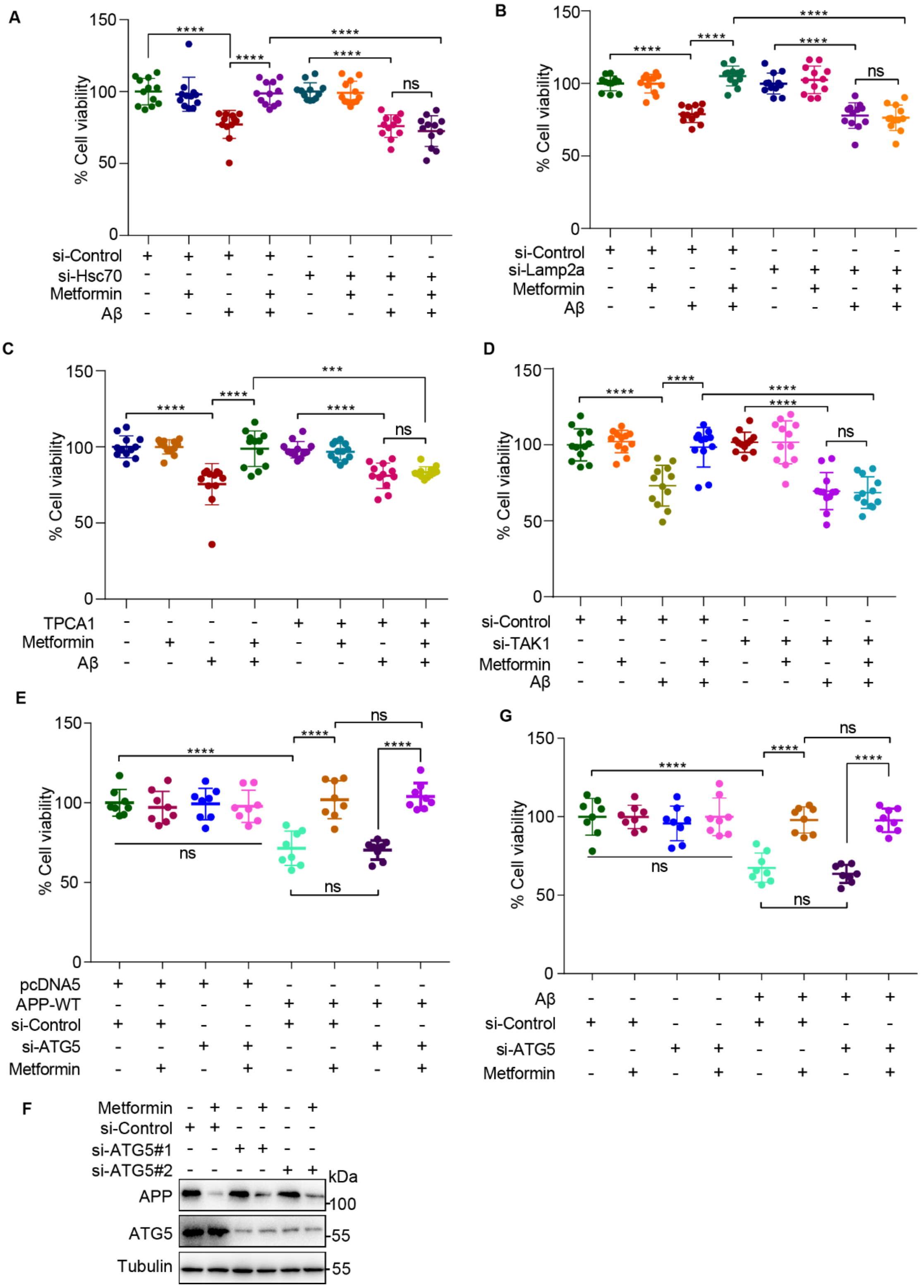

Supplementary Fig. 7 Metformin alleviates the toxicity of Aβ in an IKKα/β-dependent manner.

(**A**) PC12 cells were transfected with siRNA of Hsc70, treated with or without $A\beta_{25\text{-}35}$ (20 μM) and Metformin (20 μM) for 96 h, cell viability was determined using CellTiterGlo® assay (data represents Mean ± SD; ****p < 0.0001, one-way ANOVA).

(**B**) PC12 cells were transfected with siRNA of Lamp2a, treated with or without A$\beta_{25\text{-}35}$ (20 μM) and Metformin (20 μM) for 96 h, cell viability was determined using CellTiterGlo® assay (data represents Mean ± SD; ****p < 0.0001, one-way ANOVA).

(**C**) PC12 cells were treated with A$\beta_{25\text{-}35}$ (20 μM) and Metformin (20 μM) for 72 h, with or without TPCA1 (5 μM) for another 24 h, cell viability was determined using CellTiterGlo® assay (data represents Mean ± SD; ***p < 0.001, ****p < 0.0001, one-way ANOVA).

(**D**) PC12 cells were transfected with siRNA of TAK1, treated with or without A$\beta_{25\text{-}35}$ (20 μM) and Metformin (20 μM) for 96 h, cell viability was determined using CellTiterGlo® assay (data represents Mean ± SD; ****p < 0.0001, one-way ANOVA).

(**E**) SH-SY5Y cells were co-transfected with APP-WT and si-ATG5 or si-Control for 12 h, treated with or without Metformin (20 μM) for another 48 h, cell viability was determined using CellTiterGlo® assay (data represents Mean ± SD; ****p < 0.0001, one-way ANOVA).

(**F**) SH-SY5Y cells were transfected with si-ATG5 or si-Control for 12 h, treated with or without Metformin (20 μM) for another 48 h, cell lysates were immunoblotted with indicated antibodies.

(**G**) PC12 cells were transfected with siRNA of ATG5, treated with or without A$\beta_{25\text{-}35}$ (20 μM) and Metformin (20 μM) for 96 h, cell viability was determined using CellTiterGlo® assay (data represents Mean ± SD; **** p < 0.0001, one-way ANOVA).

Table S1. The siRNA sequences used in this study

Genes	siRNA Sequences
Hsc70#1	5'-GGCCAGUAUUGAGAUCGAUTT-3'
Hsc70#2	5'-GAGAGACCAAAAGCUUCUATT-3'
Lamp2a#1	5'-GGAGCAUUUCAGAUAAAUAUU-3'
Lamp2a#2	5'-GCAGCAUCUACUUAUUCAAUU-3'
IKKα#1	5'-GGAUGUUGGUGGAAAGAUAAU-3'
IKKα#2	5'-GGCUACAGUUGAUGUUGAAUU-3'
IKKβ#1	5'-GGAGAAGUACAGCGAGCAAAC-3'
IKKβ#2	5'-GGACAUUGUUGUUAGCGAAGA-3'
AMPKα1#1	5'-GCAGAAGUAUGUAGAGCAAUC-3'
AMPKα1#2	5'-CAAAGUCGACCAAAUGAUAUU-3'
AMPKα2#1	5'-GGAAGGUAGUGAAUGCAUACC-3'
AMPKα2#2	5'-GACAGAAGAUUCGCAGUUUUU-3'
TAK1#1	5'-GAGUGAAUCUGGACGUUUAAG-3'
TAK1#2	5'-CCUGGUACAGGAACAUAAAUU-3'
ATG5#1	5'-GCAACUCUGGAUGGGAUUGUU-3'
ATG5#2	5'-CAUCUGAGCUACCCGGAUAUU -3'
Control	5'-UUCUCCGAACGUGUCACGUUU-3'
